# Evolution of tribo-induced interfacial nanostructures governing superlubricity in a-C:H and a-C:H:Si films

**DOI:** 10.1038/s41467-017-01717-8

**Published:** 2017-11-22

**Authors:** Xinchun Chen, Chenhui Zhang, Takahisa Kato, Xin-an Yang, Sudong Wu, Rong Wang, Masataka Nosaka, Jianbin Luo

**Affiliations:** 1 State Key Laboratory of Tribology, Department of Mechanical Engineering, Tsinghua University, Beijing, 100084 China; 2 Surface Science and Tribology Laboratory, Department of Mechanical Engineering, The University of Tokyo, Tokyo, 113-8656 Japan; 3 Institute of Physics, Chinese Academy of Sciences, Beijing, 100190 China; 4 Ningbo Institute of Materials Technology and Engineering, Chinese Academy of Sciences, Ningbo, 315201 China

## Abstract

Hydrogenated amorphous carbon (a-C:H) is capable of providing a near-frictionless lubrication state when rubbed in dry sliding contacts. Nevertheless, the mechanisms governing superlubricity in a-C:H are still not well comprehended, mainly due to the lack of spatially resolved structural information of the buried contact surface. Here, we present structural analysis of the carbonaceous sliding interfaces at the atomic scale in two superlubricious solid lubricants, a-C:H and Si-doped a-C:H (a-C:H:Si), by probing the contact area using state-of-the-art scanning electron transmission microscopy and electron energy-loss spectroscopy. The results emphasize the diversity of superlubricity mechanisms in a-C:Hs. They suggest that the occurrence of a superlubricious state is generally dependent on the formation of interfacial nanostructures, mainly a tribolayer, by different carbon rehybridization pathways. The evolution of such anti-friction nanostructures highly depends on the contact mechanics and the counterpart material. These findings enable a more effective manipulation of superlubricity and developments of new carbon lubricants with robust lubrication properties.

## Abstract (web summary)

Hydrogenated amorphous carbon is a promising solid lubricant, but the underlying mechanisms surrounding its superlubricity remain unclear. Here the authors reveal that the attainment of a superlubricious state is dependent on the *in-situ*in-situ formation of a nanostructured tribolayer through different carbon rehybridization pathways.

## Introduction

Amorphous carbon (a-C), also called diamond-like carbon, exists in the form of a disordered network of *sp* ^1^, *sp* ^2^ and *sp* ^3^ bonded carbon atoms. The bonding diversity of carbon and its ability to introduce other elements into the carbon matrix allow a-C to be tailored with exceptionally well-supplied structures and properties^1^. The discovery of superlubricity in self-mated hydrogenated amorphous carbon (a-C:H) around 2000^2,3^ was the trigger that stimulated a-C as one of the most promising solid lubricants in engineering field. The friction coefficients *μ* of these films could decrease to the level of a few thousandths (i.e., *μ*~0.001), simultaneously with unmeasurable wear loss even after a long endurance test in dry inert atmosphere^3^. Since then, numerous efforts have been devoted to revealing the underlying mechanisms of superlubricity.

To date, the most prevailing theory to explain the superlubricity behaviors of a-C:Hs is surface passivation of carbon dangling bonds by hydrogen at the sliding interface^2–10^. As originally proposed for self-mated a-C:H surfaces^2–5^, the main argument is that paired C–H bonds produce repulsive forces between positively charged H atoms through partial charge transfer to C atoms. A suitable hydrogen coverage (i.e., ~ 40 at% H incorporated in the film) is therefore capable of shielding the two rubbing surfaces from direct contact and avoiding formation of covalent carbon bonds^3,4^. The contribution from the adhesive interactions to friction is therefore neglected in this case, namely without formation of a tribolayer. Nevertheless, in most other tribological experiments, the graphitization of a-C:Hs was frequently detected^11–15^, especially in the case when a-C:H is slid against an uncoated counterface. A graphitized tribolayer was formed due to the shear-induced phase transformation from *sp* ^3^-C to *sp* ^2^-C, probably as a result of hydrogen release from the a-C:H structure^11^. It has been generally assumed (without exact proof) that the produced *sp* ^2^-phase is thought to be an easy-shearing unit possessing a layered structure as graphite. Doubtlessly, hydrogen is essential to link these two anti-friction processes even though the underlying competitive relationship between them is not clear. Our recent finding of an entirely new near-frictionless behavior in self-mated Si-containing a-C:H:Si surfaces^16,17^ further emphasized the diversity and complexity of anti-friction mechanisms in a-C:Hs.

The major obstacle to disclose the atomic origins of superlubricity is that most interfacial activities occur in a very thin contact area, from which it is intractable to extract isolated signals for characterization techniques such as Raman and X-ray photoelectron spectroscopy (XPS) used in most relevant literatures^12,13,18–21^. Also, these methods are incapable of direct imaging of the interested region, which impedes the visual inspection of the sliding interface. To overcome this, a few authors have tried to detect the tribo-induced phase transformation at the sliding interface in carbon-based lubricants (i.e., diamond, a-C, a-C:H) using transmission electron microscope (TEM) and electron energy-loss microscopy (EELS)^11,22–26^. Inspiring microscopic evidence for the pathway of stressed-induced carbon hybridization during sliding has been achieved, even though some of these studies were not dealing with the research topic of superlubricious mechanisms^24–26^. Another controversy is that a large portion of the reported studies could not guarantee that the tribological experiments were conducted in a superlubricious state^20–23^, which lost the opportunities to clarify the real nature of superlubricity in a-C:Hs.

In view of these facts, we attempt to probe the tribo-induced interfacial nanostructures in several superlubricious a-C:Hs tribo-couples using the most advanced STEM and EELS after slicing a nanometer-thick lamella from the contact area by a site-specific focused ion beam (FIB). Compared with traditional TEM, STEM is a more powerful microscope with its advanced *Z*-contrast (*Z* denotes the atomic number) imaging capability at atomic-scale resolution in high-angle annular dark-field (HAADF) mode^27^. This high compositional sensitivity is particularly useful in distinguishing the elemental distribution across the tribolayer formed on heterogeneous surfaces. Along with element-analytical EELS, compositional and bonding information can be determined at high spatial resolution. It should be pointed out that only successful FIB preparation of specimens with sufficient quality for atomic resolution imaging by STEM allows this objective to be realized. The key factors concerning this FIB preparation will be published elsewhere. It is believed that such a combination of complementary methods allow the achievements of structural information of the buried interfaces at the atomic scale, making it possible to understand the relevant mechanisms of superlubricity in a-C:Hs.

## Results

### Motivation and methodology

Our starting point is to evaluate the stability of superlubricity intrinsic to hydrocarbon network by rubbing two a-C:H surfaces against each other at elevated normal loads, since contact pressure is the most critical factor affecting structural transformation upon sliding^28–30^. Any pressure-induced phase transformation should result in reconstruction of the contact interface, and perhaps the occurrence of wear (material loss). Secondly, what will occur to the friction-vanishing state when replacing one a-C:H surface by other counterpart materials such as metal or ceramic? The growth process of tribolayer along heterogeneous interface needs to be clarified. As a third concern, how is the superlubricity pathway of a-C:H affected by introducing another element into the hydrocarbon matrix, for example, the silicon-triggered hybridization dynamics and growth of a low shear-strength polymeric interface in self-mated Si-containing a-C:H:Si surfaces^16,17^? Throughout this work, special attention is paid to the structural evolution of the superlubricious tribolayer under stress and shear, especially the existence form of *sp* ^2^-C phase and the spatial distribution of hydrogen.

**Fig. 1:**
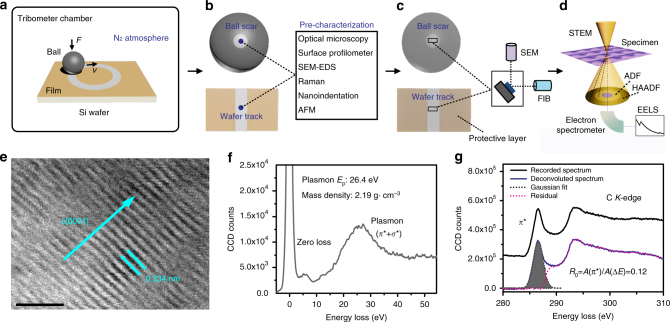
Methodology. **a**–**d** Schematic diagrams of the experiments. **a** The experimental setup for ball-on-disc friction tests in a dry N_2_ environment. **b** Various pre-characterizations of the produced contact areas including the ball wear scar and the wafer wear track after the friction tests as shown in **a**. **c** Dual beam SEM-FIB lift-out preparation of nanometer-thick lamellar specimens for TEM and STEM observations. Before FIB milling, the contact areas were protectively coated by a layer of Au or Cr with thickness measured in a few tens of nanometers. **d** STEM and EELS characterization of the FIB-fabricated lamellar specimens from the contact areas. **e**–**g** EELS quantification of highly oriented pyrolytic graphite (HOPG, 100% *sp* ^2^-C bonds) as a standard for calculations of π* and σ* bonding fractions in the C *K*-edge spectra measured from the contact area. **e** BF-STEM image showing the lamellar structure of HOPG with a lattice distance of 0.334 nm along the *c*-axis. Scale bar, 2 nm. **f** Recorded zero-loss and low-loss plasmon spectrum of HOPG. The mass density was calculated to be 2.19 g·cm^−3^ by measuring the energy *E*_p_ of the plasmon peak. **g** Recorded C *K*-edge spectrum of HOPG showing a sharp π* peak and Gaussian fit to the π* peak after background subtraction and deconvolution of the C *K*-edge spectrum. Normalizing the π* peak area to the integrated area within the energy window of 280–310 eV yields a standard value of 0.12. The measured peak ratio for a C *K*-edge spectrum referenced to this standard value finally yields the *sp* ^2^ fraction in the unknown sample

The two near-frictionless hydrogen-rich a-C:H (39.3 at% H, designated as ACF-1) and a-C:H:Si (31.9 at% H and 9.3 at% Si, designated as ACF-6) films with bilayer structures (a hard a-C:H:Si interlayer was acting as a bonding layer to substrates) and atomically smooth surfaces (Ra~0.1 nm) were synthesized by an ion vapor deposition method^16,17^, with details shown in Supplementary Fig. 1. The superlubricity behaviors were evaluated by a ball-on-disc tribometer in dry N_2_ atmosphere (Fig. 1a), and then followed by various basic and STEM/EELS characterizations of the contact areas (Fig. 1b–d). Four rubbing couples including self-mated a-C:H surfaces under normal loads of 2–10 N, bare SUJ2 steel ball vs a-C:H coated Si wafer, bare Si_3_N_4_ ball vs a-C:H coated Si wafer and self-mated a-C:H:Si surfaces were selected to investigate the influences of contact pressure, counterpart material and doping element on superlubricity in a-C:Hs. Nanoscale TEM lamellar specimens from the contact area for bright field (BF) and *z*-contrast HAADF STEM observations were fabricated using the FIB in situ lift-out technique (see Methods). For EELS quantification, highly oriented pyrolytic graphite (HOPG, 100% *sp* ^2^-C bonds) served as a standard for calculations of π* and σ* bonding fractions in the C *K*-edge spectra measured from the contact area (Fig. 1e–g). To avoid any electron-irradiation-induced artificial changes in the specimens, the operation conditions such as acquisition time during STEM and EELS observations were first optimized at a position far away from the targeted area before the formal measurements. More details are given in Methods.

### Stability of superlubricity intrinsic to hydrocarbon network

**Fig. 2:**
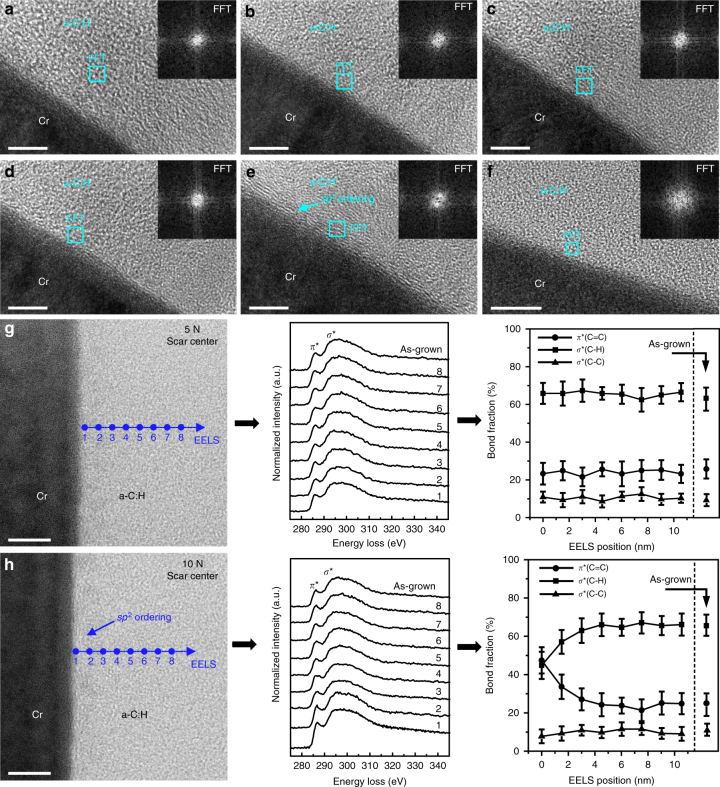
Characterization of the contact areas for self-mated a-C:H (ACF-1) surfaces. The corresponding superlubricity tests at various normal loads are shown in Supplementary Fig. 2. **a** HRTEM image of the as-grown a-C:H film on SUJ2 steel ball. (Inset) FFT image derived from the square bright-blue box (2 nm × 2 nm) shows the typical amorphous character of the film. **b**–**f** HRTEM images showing the bonding structures of the sliding interfaces from different contact areas: **b** ball wear scar center at 2 N, **c** ball wear scar center at 5 N, **d** ball wear scar edge at 10 N, **e** ball wear scar center at 10 N and **f** wafer wear track center at 10 N. (Insets) FFT images obtained from the local 2 nm × 2 nm bright-blue boxes indicate the structural evolution of the outer-most sliding interfaces. Scale bars in **a**–**e** and **f** are 5 and 10 nm, respectively. The initial peak Hertz contact pressures under loads of 2, 5 and 10 N are 0.68, 0.93 and 1.17 GPa, respectively. **g** BF-STEM image and EELS results indicating the nearly intact bonding structure of the a-C:H layer near the sliding interface for the scar center at 5 N. **h** BF-STEM image and EELS results confirming the *sp* ^2^ clustering and ordering in the outer-most ~ 3 nm region near the sliding interface for the scar center at 10 N. Scale bars in **g** and **h**, 5 nm. Error bars in **g** and **h** represent s.d. of calculated carbon bond fractions

While near-wearless sliding surfaces have long been reported for self-mated a-C:H^3,4,^, the microstructural response of hydrocarbon network subjected to the local contact stress near the surface has yet be clarified, especially on an atomic level. To this end, we first carried out friction tests to the self-mated a-C:H (ACF-1) films at various normal loads. Clearly, superlow friction was readily achieved after a quite short running-in stage (Supplementary Fig. 2a) for self-mated a-C:H surfaces. The superlubricity state depended on contact pressure, namely a higher contact pressure resulting in a lower *μ*. The steady-state *μ* are 0.008, 0.006 and 0.001, respectively, for normal loads of 2, 5 and 10 N. Smooth scar platforms without tribo-debris were produced on the a-C:H coated SUJ2 ball surfaces, and simultaneously nearly invisible wear tracks were found on the a-C:H coated Si wafers (Supplementary Fig. 2b). The wear depth was unmeasurable for loads up to 5 N (Supplementary Fig. 2c), and a maximum of ~ 11 nm was detected for 10 N (Supplementary Fig. 2d). TEM images (Supplementary Fig. 3a–e) clearly show that the overall thickness of the film in the scar region gradually decreased with the increase in contact pressure, implying that the bulk of the film extended laterally to form the platforms. Figure 2 presents the bonding structures of the sliding interfaces under various contact pressures. For loads up to 5 N, the film structures near the top-most contact zones remained intact, as confirmed by the FFT (Insets in Fig. 2a–c), Raman (Supplementary Fig. 2e) and EELS results (Fig. 2g). However, for the load of 10 N, clustering and local ordering of *sp* ^2^-C phase in the outer-most ~ 3 nm region of the central scar surface was observed, as shown in Fig. 2e, h. This shear region had a higher fraction of *sp* ^2^-C phase as compared to the as-grown bulk film (47.5% vs 25%), meanwhile maintaining a high density of *sp* ^3^(C–H) bonds (45% vs 65% for the as-grown film). The bonding structure of this anti-friction shear band is more like a highly-hydrogenated graphite-like carbon (GLCHH)^31,32^. For a ball-on-disc contact geometry, the distribution of contact pressure is spatially non-uniform, where the scar center always suffers higher stress concentration than the scar edge^33^. This was verified by noticing the nearly intact bonding structure of the contact interface from the scar edge area at 10 N (Fig. 2d). In addition, the film structure of the counterpart a-C:H surface on Si wafer remained intact even for the sliding condition at 10 N (Fig. 2f). Clearly, the self-mated hydrocarbon network could at least bear a peak contact pressure of 0.93 GPa to yield a near-frictionless state without any bonding fracture and material loss, while tribo-induced structural transformation and nanoscale wear occurred at a pressure up to 1.17 GPa. In the steady state (Supplementary Fig. 2a, b), the calculated shear strength of the interfaces^34^ at the loads of 2, 5 and 10 N were 2.16, 2.47 and 0.62 MPa, respectively. The noticeable reduction of shear strength at 10 N also implied the possible occurrence of pressure-induced structural change in the rubbing region, as observed above.

### Growth of nanoscale tribolayers on heterogeneous surfaces

**Fig. 3:**
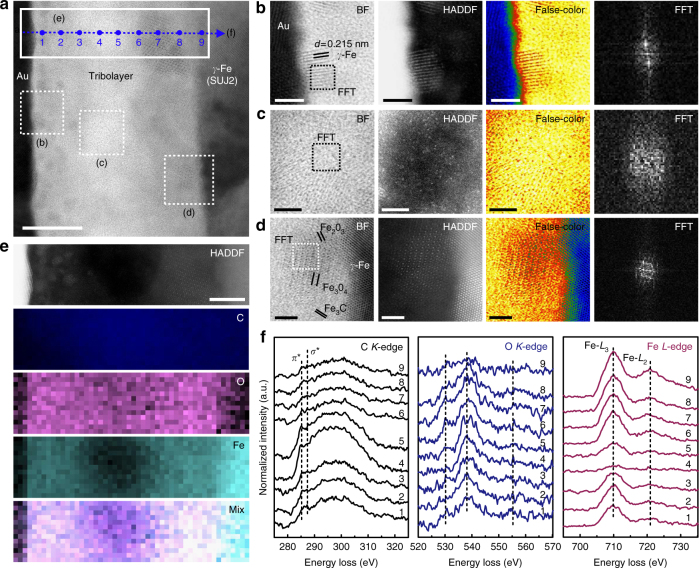
Characterization of the contact area produced on bare SUJ2 steel ball surface. The corresponding superlubricity test paired with a-C:H (ACF-1) surface is shown in Supplementary Fig. 4. **a** BF-STEM image showing a carbonaceous tribolayer of ~ 27 nm thickness in situ generated on the steel ball surface. The tribolayer internally evolved into three individual sublayers (marked as **b**, **c** and **d**): a Fe-nanoparticle-dispersed sublayer near the sliding interface, a C-rich low density sublayer in the middle and a C-Fe-O heterogeneously well-crystalized sublayer near the steel ball surface. Scale bar, 10 nm. **b**–**d** BF-, HAADF-, false-color displayed BF-STEM and local FFT images of the three sublayers as marked in **a**. Scale bars in **b**–**d** are all 2 nm. **e** HAADF-STEM image showing the EELS spectrum-image (SI) mapping area as marked in **a**. The corresponding EELS C-*K*, O-*K*, Fe-*L* elemental maps and their composite are presented. Scale bar, 5 nm. **f** Evolution of *C-K*, O-*K* and Fe-*L* EELS core-edge spectra recorded across the tribolayer as marked in **a**

As observed above, the superlubricious state between self-mated a-C:H surfaces was unambiguously stable at a quite high level of contact stress, even in some instances this occurred in conjunction with the formation of easy-shearing interfacial nanostructures beyond a critical shear stress. To access the possibility of achieving a near-frictionless state between heterogeneous surfaces, we replaced one a-C:H (ACF-1) surface by bare SUJ2 steel ball. As shown in Supplementary Fig. 4a, the superlubricious state appeared after a prolonged running-in stage, finally achieving a stable superlow *μ* of 0.004. After test, a dark-brown worn scar was produced on the ball surface, while the wear track on a-C:H surface was barely visible to naked eyes with wear depth below 7 nm (Supplementary Fig. 4b, c). Raman spectra indicated the remarkable increase and local clustering (formation of six-fold aromatic rings)^32^ of *sp* ^2^-C phase in the scar region (Supplementary Fig. 4d). TEM and STEM images (Fig. 3a and Supplementary Fig. 5a) confirmed the formation of a uniform carbon-rich tribolayer of ~ 27 nm thickness on the ball scar surface. The tribolayer was composed of three individual sublayers including a Fe-nanoparticle-dispersed sublayer near the sliding interface (Fig. 3b), a C-rich low density sublayer in the middle (Fig. 3c) and a C–Fe–O heterogeneously crystalized sublayer close to the steel surface (Fig. 3d). The overall morphology of this layered structure is more easily distinguished in a false-color displayed HAADF image as shown in Supplementary Fig. 5c. The EDS-elemental distribution across the tribolayer (Supplementary Fig. 5b, d) confirmed the layered structure. The chemically bonding states of the tribolayer were further examined by EELS analysis. Figure 3e shows the EELS elemental maps of C, Fe and O, verifying again the spatial distribution of interfacial atoms mentioned above. Figure 3f presents the EELS spectrum image (SI)-line spectra of C-*K*, O-*K* and Fe-*L* core edges recorded point by point across the tribolayer, revealing the evolution of bonding environment for each element. Clearly, carbon appeared to be active in forming a *sp* ^2^-C phase dominating region in the middle of the tribolayer as the sharpening π* peaks in the C-*K* edges (EELS points 4 and 5) imply. The calculated *sp* ^2^-C fraction of this sublayer was in the range of 65–70% (Supplementary Fig. 5f, g), with the residual fraction assigned to σ* bonding (20–25% for *sp* ^3^(C–H) and ~ 10% for *sp* ^3^(C-C)). Furthermore, the mass density of this region, estimated from the plasmon energy in the low loss spectrum (Supplementary Fig. 5e), was around 1.53 g cm^−3^ or even lower (cf. 2.27 g cm^−3^ of graphite). From the Raman spectrum (Supplementary Fig. 4d), STEM images and FFT result (Fig. 3c), it was inferred that these *sp* ^2^-C phases existed mainly in amorphous form, but with nano-clusters ordered locally. From O-*K* and Fe-*L* edges, it is expected that the tribolayer was bonded to the steel surface through the formation of iron oxides and iron carbides near the steel surface (EELS points 6–8). The influence of oxygen adsorption on the characterization results is discussed in Methods. Besides, it is interesting to note that iron gathered at the outer-most sliding interface in the form of nanoparticles, probably through shear-induced diffusion from the steel surface (EELS points 1–3). At present, the exact role of these Fe nanoparticles is not clearly understood yet. It is highly possible that they were supposed to passivate the outer-most shear band since they could remain in this anti-friction interface until the end of the tribological test. Simultaneously, this region was particularly rich in hydrogen (~58% *sp* ^3^(C–H) in C-*K* edge, Supplementary Fig. 5g). The concentration of hydrogen further passivated the carbonaceous tribolayer, producing a chemically inert surface. On the counterpart side (Supplementary Fig. 5h, i), it is surprising to observe that the bonding structure of the a-C:H surface near the sliding interface was nearly intact after the friction test even though ~ 7 nm thick film material had been transferred to the steel ball surface. Apparently, the prolonged running-in stage was mainly involved in growth of such a nanostructured tribolayer on the bare steel surface to establish the superlubricity state.

**Fig. 4:**
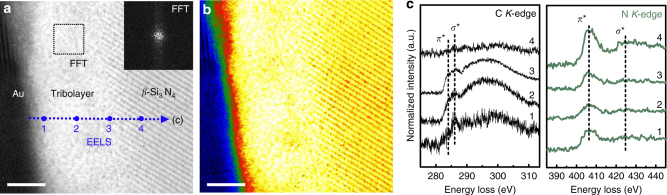
Characterization of the contact area produced on bare Si_3_N_4_ ball surface. The corresponding superlubricity test paired with a-C:H (ACF-1) surface is shown in Supplementary Fig. 6. **a** BF-STEM image showing a carbonaceous tribolayer of ~ 5 nm thickness in situ generated on the ceramic ball surface. (Inset) FFT image derived from the square dashed box demonstrating the mainly amorphous nature of the formed tribolayer. **b** The corresponding false-color displayed BF-STEM image for **a**. Scale bars in **a** and **b**, 2 nm. **c** Evolution of C-*K* and N-*K* EELS core-edge spectra recorded across the tribolayer as marked in **a**

To further examine whether such an anti-friction tribolayer grows on a ceramic surface, we performed friction tests by sliding a-C:H (ACF-1) surface against bare Si_3_N_4_ ball. Clearly displayed in Supplementary Fig. 6a, the superlubricious state was also quickly established without a long running-in stage, obtaining a stable *μ* of 0.004. After friction test, optical images show that almost no tribo-material was found in the central region of the produced wear scar on ball, while some debris piled up on the edge of the scar (Supplementary Fig. 6b). On the other side, the wear track on a-C:H surface was nearly visible to naked eyes with wear depth below 7 nm (Supplementary Fig. 6b, c). However, Raman analysis implied the existence of plentiful *sp* ^2^-C phases both in the central region of the scar and on the edge (Supplementary Fig. 6d). Note that the local clustering degree of these *sp* ^2^-C phases was weaker as compared to the bare SUJ2 steel counterpart by noticing the shape variation of the *D*-peaks^32^ (Supplementary Fig. 6d vs Supplementary Fig. 4d). STEM images (Fig. 4a, b) confirmed the in situ growth of a uniform carbonaceous tribolayer of ~ 5 nm thickness on the central Si_3_N_4_ scar surface. The bonding interface was sharp, and no obvious layered structure was observed in the tribolayer. The main composition of the tribolayer was carbon with a fraction of 65–80 at% (Supplementary Fig. 7b, c), forming an amorphous matrix (FFT result, inset in Fig. 4a) with a density of about 1.66 g cm^−3^ (Supplementary Fig. 7d). The presence of trace elements such as N and Si (Supplementary Fig. 7c) and the recorded N-*K* edges (Fig. 4c) imply that the Si_3_N_4_ surface was involved in tribochemical reactions to some extent in spite of its chemical inertness and high hardness. By fitting the C-*K* edges (Fig. 4c and Supplementary Fig. 7g), we can obtain some bonding information in the tribolayer. The fraction of *sp* ^2^-C phase in the main body of the tribolayer was ~ 80% (EELS point 2), while *sp* ^3^(C–H) bonds were preferentially formed near the sliding interface (up to 40%, EELS point 1). For the counterpart a-C:H surface (Supplementary Fig. 7h, i), the bonding structure of the outer-most region was once again nearly intact after the superlubricity test, resembling the above two rubbing couples.

### Si-triggered tribo-softening of sliding interface

**Fig. 5:**
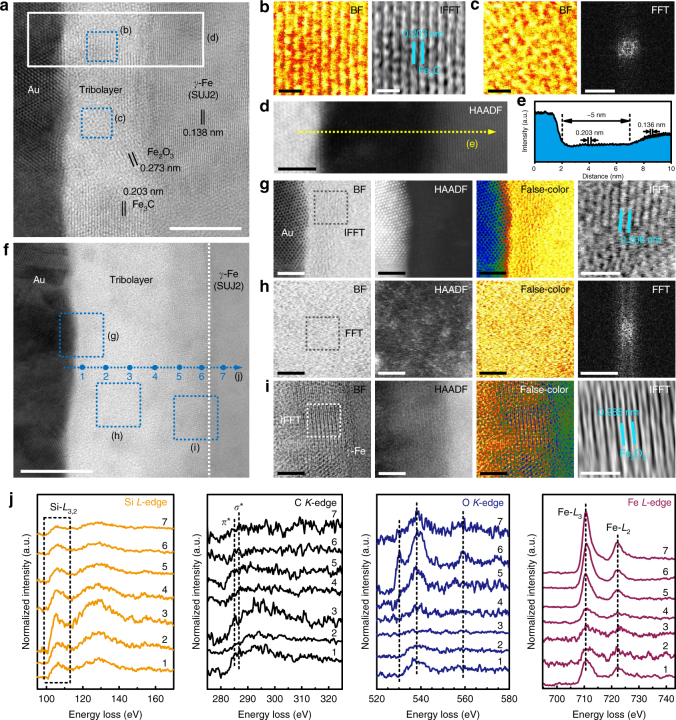
Characterization of the contact area produced on a-C:H:Si film-coated SUJ2 steel ball surface. The corresponding superlubricity test for self-mated a-C:H:Si (9.3 at% Si, ACF-6) surfaces is shown in Supplementary Fig. 8. The transfer of all the film material from the ball surface to the wafer side and the simultaneous re-generation of tribolayers on both the surfaces demonstrate the reconstruction of the sliding interface (Supplementary Figs 8 and 9). **a** BF-STEM image showing a mainly C-Fe intermixed tribolayer of ~ 5 nm thickness generated at the ball wear scar center. Scale bar, 5 nm. **b**, **c** BF-STEM and IFFT (or FFT) images specifying a highly-crystalized Fe_3_C shear band (**b**) and an amorphous-like local structure (**c**) in the tribolayer as marked in **a**. Scale bars in **b**, 0.5 nm. Scale bars of BF and FFT images in **c** are 0.5 nm and 20 nm^−1^, respectively. **d** HAADF-STEM image across the tribolayer as marked in **a**. Scale bar, 2 nm. **e** Intensity profile along the dashed line as marked in **d** confirming the atomic structure of the tribolayer. **f** BF-STEM image showing a tribolayer of ~ 20 nm thickness generated on the ball wear scar edge. Scale bar, 10 nm. The tribolayer internally evolved into three individual sublayers (marked as **g**, **h** and **i**): a well-crystalized Fe-rich sublayer near the sliding interface, a C-rich amorphous sublayer in the middle and a highly oxidized Fe-based sublayer near the steel ball surface. **g**–**i** BF-, HAADF-, false-color displayed BF-STEM and local IFFT (or FFT) images of the three sublayers as marked in **f**. Scale bars of BF-, HAADF-, false-color displayed BF-STEM images in **g**–**i** are all 2 nm, while scale bars of IFFT (FFT) images in **g**–**i** are 1 nm, 20 nm^−1^ and 1 nm, respectively. **j** Evolution of Si-*L*, C-*K*, O-*K* and Fe-*L* EELS core-edge spectra recorded across the tribolayer as marked in **f**

In contrast to the case of near-frictionless a-C:H discussed above, our recent study^17^ found that the anti-friction behavior of the self-mated a-C:H:Si (ACF-6) surfaces was unprecedented after introducing Si into the a-C:H matrix. As illustrated in Supplementary Fig. 8a, an extremely low *μ* of 0.001 was also rapidly realized at the onset of sliding contact. However, being different from the above three couples, the as-deposited film materials on the ball surface were all transferred to the counterpart a-C:H:Si surface in the running-in stage^17^, namely in situ growth of a tribolayer in the wear track. Consequently, a relatively smooth worn scar was generated on the SUJ2 ball surface (Supplementary Fig. 8b, c), whereas a quite uneven wear track covered by a thick tribolayer was observed on the wafer side (Supplementary Fig. 8b, d). This tribolayer had a quite low hardness (~ 0.25 GPa, Supplementary Fig. 8e), resembling that of a hydrocarbon polymer^35^. Raman results (Supplementary Fig. 8f) demonstrated the enhancement of *sp* ^2^-phase in the contact areas including both the ball worn scar and the wafer wear track. TEM and STEM observations further confirmed the presence of site-specific nanoscale tribolayers on the scar surface. In the central region of the scar (Supplementary Fig. 9a), a highly crystallized and ordered tribolayer with thickness of ~ 5 nm was in situ generated (Fig. 5a, d, e and Supplementary Fig. 9b). The main body was Fe_3_C (Fig. 5b), simultaneously intermixed with some amorphous phases (Fig. 5c). This tribolayer was speculated to act as a shear band to passivate the contact surface. On the scar edge (Supplementary Fig. 9c), a thicker tribolayer of ~ 20 nm was present, possessing a more subtle bonding structure (Fig. 5f). Being similar to the case of bare SUJ2 steel ball, the produced tribolayer was composed of three sublayers: namely, a well-crystalized Fe-rich sublayer near the sliding interface (Fig. 5g), a C-rich amorphous sublayer in the middle (Fig. 5h) and a highly oxidized Fe-based sublayer near the steel ball surface (Fig. 5i). This layered structure is more vividly displayed in a HAADF image (Supplementary Fig. 9e), and further verified by the EDS-elemental distribution across the tribolayer (Supplementary Fig. 9d, f). The crystal phases in the outer-most sublayer were detected to be Fe_3_C and Fe (lattice constant of 0.206 nm, IFFT result in Fig. 5g), with their shear layers oriented parallel to the sliding direction. The middle sublayer had a carbon fraction up to ~ 71 at%, yielding a mass density of about 1.89 g cm^−3^. The tribolayer was tightly bonded to the steel surface via in situ formation of oxide compounds such as Fe_2_O_3_ (Fig. 5i). The evolution of EELS Si-*L*, C-*K*, O-*K* and Fe-*L* edges recorded across the tribolayer provides more delicate evidence to support the declared layered structure. Special attention should be paid to the distribution of Si atoms in the tribolayer. It was found that the concentration of Si near the outer-most scar surface was lower, as compared to the main body of the tribolayer (Supplementary Fig. 9f). Similar trend could be found in the wear track (Supplementary Fig. 9i). The role of Si in establishing the anti-friction interface is discussed below. As for the counterpart a-C:H:Si surface on Si wafer, the TEM cross-sectional image shows the interfacial waviness along the sliding wear track (Supplementary Fig. 9g), confirming the rough morphology of the tribolayer. A top-most region of ~ 30 nm in the wear track was found to undergo harsh phase transformation (Supplementary Fig. 9h, i). The carbon fraction could reach up to ~ 75 at%, whereas the Si concentration near the sliding interface was low (~ 7 at% vs ~ 22 at% for the bulk). The EELS analysis further clarified the bonding evolution in the tribolayer, as shown in Supplementary Fig. 9j, k. The transformation region had a higher fraction of *sp* ^2^-C phase (up to ~ 57%), meanwhile retaining a high density of σ*-hybridized bonds (i.e., C–H, C–Si, C–C). Taking the low hardness into account, the bonding structure of the tribolayer was therefore more like a hydrocarbon polymeric compound. This new phenomenon found in tribo-induced carbon hybridization processes, namely Si-triggered tribo-softening of the sliding interface, is a supplement to the superlubricity mechanisms in a-C:H. Apparently, the corresponding structural transformation process and its energetic pathway is closely related to the doping effect of Si on the hydrocarbon lubrication matrix.

**Fig. 6:**
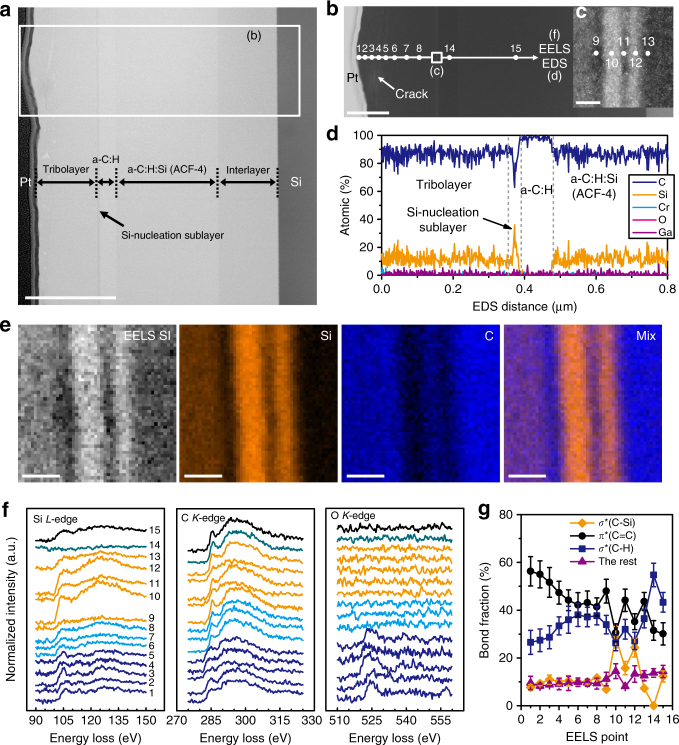
Characterization of the contact area produced on a-C:H:Si film-coated Si wafer surface. The corresponding friction test for self-mated a-C:H:Si (5.7 at% Si, ACF-4) surfaces is shown in Supplementary Fig. 10. The tribo-induced transfer of partial film material from the ball surface to the wafer side and the Si-triggered growth of an incomplete tribolayer in the wafer wear track were observed. Note that, to distinguish the growth interface of the tribolayer on the wafer side, a pure a-C:H layer was deposited on top of the a-C:H:Si (ACF-4) layer. **a** BF-STEM image showing the cross-sectional morphology of the tribolayer-covered wafer wear track. The growth of this tribolayer was triggered following the formation of a Si-nucleation sublayer along the sliding interface. Scale bar, 0.5 μm. **b** HAADF-STEM image across the wafer wear track as marked in **a**. Scale bar, 0.2 μm. **c** HAADF-STEM image showing the Si-nucleation sublayer. Scale bar, 10 nm. **d** EDS-elemental distribution across the wafer wear track as marked in **b**. **e** EELS-SI mapping image of the Si-nucleation layer and the corresponding extracted Si-*L*, C-*K* and mix maps. Scale bars, 10 nm. **f** Evolution of Si-*L*, C-*K* and O-*K* EELS spectra recorded across the cross-sectional wear track as marked in **b** and **c**. **g** Evolution of the calculated C-bonds fractions from the C-*K* edges presented in **f**. Error bars denote s.d. of calculated carbon bond fractions

This critical finding further prompted us to answer the question what specific role of Si atoms was involved in growing such an anti-friction tribolayer. To explore the underlying mechanism, we synthesized another four groups of hydrogen-rich a-C:H:Si films (designated as ACF-2 to ACF-5) by gradually increasing the Si content from ~ 0 at% (ACF-1) to 9.3 at% (ACF-6), as detailed in Supplementary Fig. 10a. The tribological tests (Supplementary Fig. 10b) clearly verify the strong dependence of anti-friction behaviors in a-C:H:Si films on the Si content, and superlow friction is more feasible when the Si content is below 4.2 at% or higher than 8.5 at%. Moreover, a ‘bump-like’ high friction running-in stage (Supplementary Fig. 10c) appeared for all the a-C:H:Si films. The most interesting observation is the evolution of tribolayer coverage on the wafer side with respect to the Si content as shown in Supplementary Fig. 10d, namely, from the absence of tribolayer (~ 0 at% Si) to the development of an incomplete tribolayer (4.2–8.5 at% Si), then to the formation of a fully complete tribolayer (9.3 at% Si). The growth of tribolayers in self-mated a-C:H:Si films was always coupled with the film material transfer from the ball side to the wafer side. It seems that the complete covering of the wear track by a uniform tribolayer is the necessity for achieving a near-frictionless state (i.e., *μ*~0.001 for ACF-6). Obviously, the atomic-scale construction of the initial contact interface should play a pivotal role in triggering the following growth of the tribolayer. To distinguish it, we tried to separate the grown tribolayer from the underlying a-C:H:Si layer by depositing a Si-free a-C:H layer on top of the a-C:H:Si layer. The sample ACF-4 (5.7 at% Si) was selected for this purpose. Figure 6 and Supplementary Fig. 11 show the STEM and EELS characterization results of the targeted specimens sliced from the contact area by FIB (Supplementary Fig. 10d). In the wear track, a thick tribolayer was formed on top of the Si-free a-C:H layer (BF-STEM image, Fig. 6a). It was surprising to find that a nanoscale sublayer exists on the bottom of the tribolayer, namely the initial starting point for the tribolayer growth. The overall morphology of the tribolayer is more clearly distinguished in a HAADF image as shown in Fig. 6b. It consists of a bottom sublayer, a dense middle-layer and a loose top-layer with some cracks and pores. The bottom sublayer was detected to be a Si-nucleation layer with Si content up to 37 at%, as confirmed by the high-magnification HAADF image (Fig. 6c) and the EDS result (Fig. 6d). The bonding structure of the Si-nucleation sublayer was further resolved by EELS-SI mapping characterization as displayed in Fig. 6e. The obtained SI image and the corresponding extracted Si-*L*, C-*K* and mix maps clearly show the slight fluctuation of Si and C concentrations within the Si-nucleation sublayer. Figure 6f further presents the evolution of EELS Si-*L*, C-*K* and O-*K* edges recorded point by point across the cross-sectional wear track, which provides more accurate bonding information for the grown tribolayer. One noticeable point is the alternative variations of the ionization edge shapes in Si-*L* and C-*K* spectra (Points 9–13), in accordance with the SI results (Fig. 6e). Another finding is the detection of oxygen adsorption in O-*K* edges (Points 1–5), which confirms the loose and porous structure of the tribolayer in the near-surface area. The calculated C-bonds fractions from the C-*K* edges are summarized in Fig. 6g. Being similar to the case of self-mated a-C:H:Si (9.3 at% Si), the near-surface region had a higher fraction of *sp* ^2^-C phase (up to ~ 56%), meanwhile retaining a high density of σ*-hybridized bonds (i.e., C–H, C–Si). On the ball side, however, we found that not all the ACF-4 layer was transferred to the wafer side (Supplementary Fig. 11a), implying an incomplete tribo-hybridization progress at the present Si content. High-magnification BF-STEM image and EDS results (Supplementary Fig. 11b, c) also detected the formation of a tribo-affected near-surface area, namely a tribolayer, in the remaining ACF-4 layer. The EELS results (Supplementary Fig. 11d, e) once again confirmed the near-surface region of the tribolayer was highly *sp* ^2^-bonded and simultaneously possessed a notable fraction of *sp* ^3^-hybridized bonds.

## Discussion

Now we discuss the superlubricity mechanisms in more detail. The above results demonstrate a general standpoint that the occurrence of a superlubricious state in a-C:Hs is always coupled with the establishment of an anti-friction nanostructure at the sliding interface. It is capable for a-C:Hs to achieve superlow friction in a wide range of tribological conditions, generally via in situ growth of a nanoscale carbonaceous tribolayer in the contact area. Running-in is a critical aspect to affect this process. A number of tribochemical reactions such as bond cleavage, migration and re-arrangement of interfacial atoms and bond-formation, are thought to occur during this period^36–38^. For self-mated a-C:H surfaces, a-C:H film on the ball side accommodated the initial high contact pressure through extending its bulk laterally instead of fracture-induced material loss. It is competent for the paired a-C:Hs to lubricate themselves by the chemically inert hydrogen-passivated surfaces^3–5^ under an initial peak pressure up to 0.93 GPa without any detectable wear loss (Supplementary Fig. 2b, c). Nevertheless, nanoscale material loss occurred when the contact pressure further increased to 1.17 GPa (Supplementary Fig. 2b, d). Hydrogen atoms were considered to be cleaved from the local highly-stressed C–H asperities, leaving C atoms re-hybridized into *sp* ^2^-C phase^39^, even though in most situations C–H bonding is very strong and remove of hydrogen from carbon is quite difficult. To retain the weak interactions between atoms, these *sp* ^2^-C clusters were deeply ordered in the very thin shear layer (~ 3 nm) with most of their bonds orienting parallel to the sliding direction (Fig. 2e, h). This shear-induced localization and ordering phenomenon was also observed in MD simulations^30,37^. Note that this region was still rich in hydrogen, i.e., about 45% of the C-bonds being σ*(C–H) bonded. Therefore, the bonding configuration was preferentially regarded as GLCHH^31^. This low-strength shear band further canceled out the interfacial friction forces, lowering *μ* to an extremely value of ~ 0.001. This is also the level of friction-vanishing recorded in the literature^3,4^. When replacing one a-C:H surface by the bare steel ball, it required a noticeable running-in stage (Supplementary Fig. 4a) to build up the near-frictionless tribolayer owing to the high reactivity of iron and the existence of pristine oxide layers on its surface^7^. At the onset of sliding contact, hydrocarbon fragments from a-C:H surface made bonds with the oxidized steel surface to form a highly-crystallized C-Fe-O bonding sublayer (Fig. 3d), then followed by the growth of the other sublayers with high fraction of *sp* ^2^-C phase (Fig. 3b, c). Note that hydrogen loss in the phase transformation of *sp* ^3^-to-*sp* ^2^ during the running-in period is sometimes significant, i.e., in the form of mechanically stimulated gas emission^40^. With the proceeding of sliding contact, shear-induced layering and ordering further reconstructed the bonding environment in the tribolayer. The aggregation of Fe-based nanoparticles (i.e., Fe, Fe_3_C) at the outer-most interface tended to passivate the contact surface not only by itself, probably via producing positively charged sites through electron transfer^41^ from Fe to adjacent C atoms, but also by hydrogen enriched in this region, since a shear layer with extremely high fraction of *sp* ^2^-C (up to ~ 70%, Supplementary Fig. 5g) alone could not guarantee a superlow friction in dry N_2_ atmosphere as the pure graphite behaved^42^. Once interfacial atom arrangements within or between the paired surfaces were nearly completed and the number of atomic bonds fractured and reformed at the same time reached an extraordinarily low level, a superlubricious state between passivated surfaces was expected to appear. As for the bare Si_3_N_4_ counterpart ball, the higher chemical stability and mechanical hardness of the counterpart surface largely shortened the running-in stage (Supplementary Fig. 6a), expressed by the formation of an atomically sharp bonding interface and a much thinner tribolayer (~ 5 nm, Fig. 4a). Compared with the bare steel ball, the formed tribolayer underwent a deeper transformation under higher contact pressure (fraction of *sp* ^2^-phase up to ~ 80%, Supplementary Fig. 7g), and possessed less local ordering (Fig. 4a and Supplementary Fig. 6d). In spite of the high density of π*-bonds, plentiful hydrogen still remained in the tribolayer, especially in the region close to the sliding interface (Supplementary Fig. 7g). From the perspective of energy, the sliding process is always accompanied by energy dissipation in different pathways. The occurrence of a superlubricious state implied an extremely low level of energy dissipation between rubbing surfaces. It is hence reasonable to speculate that energy-based interfacial events such as bonds fracture and re-formation, which were strongly correlated with the production of frictional forces, should be highly suppressed. Therefore, the formation of anti-friction interfacial nanostructures, especially a newly-grown tribolayer with specific composition, was expected to occur during the running-in stage rather than in the superlubricious state. A relatively high level of frictional energy, expressed by a high *μ* in the running-in stage (Supplementary Figs. 2a, 4a, 6a, 8a and 10c), was required to facilitate this process. The energy amount consumed for driving the structural transformation of the interfacial region, namely the intensity and duration of the running-in stage, depended on the tribo-couple material and the contact mechanics. For instance, the self-mated a-C:H:Si sliding at 2 N required ~ 50 cycles with an average running-in *μ* of 0.6 to grow an complete near-frictionless tribolayer (Supplementary Figs. 8a, b and 10c), whereas the self-mated a-C:H rubbed at 10 N only needed ~ 25 cycles with initial *μ* < 0.05 to establish a superlow friction state (Supplementary Fig. 2a).

When introducing Si into the hydrocarbon matrix, the modified bonding structure provided another novel pathway to achieve superlubricity for self-mated a-C:H:Si surfaces. Namely, reconstruction of the sliding interface was realized through transfer of most film materials coated on the ball side to the counterpart a-C:H:Si surface, and simultaneously by in situ Si-triggered re-growth of anti-friction tribolayers on the two rubbing surfaces. This was inspired by the assumption that silicon might stimulate the phase transformation process during the dry sliding contact, since Si is well-known for its role in promoting the formation of a nanoporous microstructure in amorphous Si_1−*x*_C_*x*_:H alloys^43^. These nanopores were usually stress-concentrated points. Upon sliding contact, numerous crack propagations occurred, which triggered the plastic deformation and phase transformation in the a-C:H:Si layer. The tribo-softened and fractured materials were then transferred to the counterpart a-C:H:Si surface, yielding a polymeric tribolayer (Supplementary Figs. 8e and 9k). It was speculated that the formation of a Si-nucleation sublayer along the initial sliding interface was probably as a result of gas emission of hydrocarbon species (i.e., CH_4_, C_2_H_2_) from the contact asperities during the structural transformation process. Finally, smooth rubbing occurred between a highly-passivated surface (i.e., C–H, Fe_3_C and Fe shear bands, Fig. 5a–g) and a polymeric-tribolayer-covered surface, resulting in extremely low interfacial interactions and hence the appearance of a superlubricity state.

The present work along with previous studies both demonstrate that tribo-induced carbon rehybridization of *sp* ^3^-to-*sp* ^2^ is a prevailing phenomenon in carbon-based lubricants, not only for various tribo-couples such as diamond/diamond^24,28,29^, nanocrystalline diamond (NCD)/NCD^26^, tetrahedral amorphous carbon (ta-C)/ta-C^44^, tungsten/a-C:H^22,23^ and diamond/a-C^25^, but also in various ambient conditions including dry N_2_ atmosphere^13,17,21,24^, ultra-high vacuum (UHV)^2,7,22,23,40,44^, glycerol or water vapor-purged UHV^26^ and humid air^11–13,20,25^. It is emphasized that the formation of a *sp* ^2^-C rich amorphous carbon tribolayer is a quite universal mechanism governing the friction and wear behaviors of carbon coatings. However, in all these attempts, robust superlow friction (*μ* < 0.01) was only achieved for the a-C:H film^2–4,7,21,40,45^, which highlighted the pivotal role of hydrogen in anti-friction events. In highly-*sp* ^3^ bonded and H-deficient materials such as diamond, NCD and ta-C, continuous amorphization of the sliding interface under stress and shear through *sp* ^3^-to-*sp* ^2^ transformation is the origin of high friction and severe wear^24,26,29,46^, if these chemically-active rubbing asperities are not saturated by some terminating species such as H- or OH- groups^44,47^. For a-C:H lubricant, on one hand, the formation of a nanoscale *sp* ^2^-C-rich soft tribolayer with some local ordering (i.e., Fig. 3a–d) first guarantees the easy-shear capability; on the other hand, the existence of sufficient hydrogen in the tribolayer, especially the preferential aggregation in the near-surface area (i.e., Supplementary Fig. 5g), further allows the shielding of any chemical interactions between contact asperities. Together the two aspects enable the occurrence of a near-friction state in a-C:H lubricated contact. In comparison, the doping of Si into hydrocarbon matrix induced an intensified plastic deformation and a more deep structural transformation by triggering the growth of a more ‘bulk-like’ rather than nanoscale tribolayer. The Si-nucleation sublayer formed upon the initial contact was acting more like a catalytic agent, which stimulated the following growth of the tribolayer (Fig. 6a–e). The formation of a hydrogen-rich chain-developed polymeric tribolayer with low-density and superior flexibility therefore satisfied the prior condition for producing superlubricity. However, the completeness of the tribolayer profoundly affected the decreasing level of friction. As recorded (Supplementary Fig. 10b–d), the more complete the tribolayer was, the more extensive was the friction vanishing. For example, superlow friction was more likely to appear for a rubbing state between a fairly complete tribolayer and a highly-passivated surface (Fig. 5, Supplementary Figs. 8 and 9).

In summary, we have successfully probed the superlubricious interfaces in a-C:H and a-C:H:Si at the atomic scale, using FIB lift-out slicing method combined with STEM and EELS microscopic characterizations. The present work unequivocally clarifies the critical role of in situ reconstruction of the sliding interface in the establishment of a superlubricious state. The achievements of atomic features in tribo-induced interfacial nanostructures provide a deeper understanding of the anti-friction behaviors. The results demonstrate the complexity and diversity of superlubricity mechanisms in hydrocarbon-based lubricants, depending on the tribological condition. The realization of superlubricious states in various frictional cases, the straightforward manner in which the interfacial atoms arranged, and the ubiquity of associated elements (i.e., C, H, Fe, Si) in the reconstructed sliding interfaces, enable the possibilities of engineering such near-frictionless carbon-based lubricants in general. Moreover, one may extend the revealed fundamentals to a new direction to develop novel and robust properties for other surface and interface materials.

## Methods

### Synthesis of amorphous carbon films

Two types of amorphous carbon films, including hydrogenated amorphous carbon (a-C:H) and silicon-containing hydrogenated amorphous carbon (a-C:H:Si), were synthesized for superlubricity experiments. Both of them were prepared using an ion vapor deposition system. Relevant details about the system setup and growth procedure have been reported elsewhere^16,17^. In general, the films were designed to have a bilayer structure. A hydrogen-deficient and hard a-C:H:Si interlayer was first deposited on the substrates (Si wafer or SUJ2 steel ball) to act as a bonding layer. Note that mirror-polished n-type Si(100) wafers with *R*a = 0.12 nm were always used as the disc-side in the ball-on-disc friction experiment, while SUJ2 bearing steel balls were only coated for self-mated sliding. Then, hydrogen-rich a-C:H or a-C:H:Si layers were deposited on top of the interlayer to act as a sliding layer. Supplementary Figure 1a, b depicts the microstructures of these films. As observed, both a-C:H and a-C:H:Si top layers were uniformly grown on the interlayer surface. Electron diffraction pattern images demonstrate the typical amorphous bonding structures of them. Meanwhile, these amorphous films maintain the atomically smooth surfaces (*R*a~0.1 nm) as with the mirror-polished Si(100) wafers, which are confirmed by the atomic force microscopy (AFM) recorded morphologies as shown in Supplementary Fig. 1a, b. The elemental composition and mechanical properties of these films are summarized in Supplementary Fig. 1c. As indicated, the hydrogen contents of the as-grown a-C:H and a-C:H:Si films are 39.3 and 31.9 at.%, respectively. This level of hydrogen content in amorphous carbon is considered to be high, and the film bonding structure tends to be more polymer-like than diamond-like^31^, as most of the *sp* ^3^-bonds formed in the films are hydrogen-terminated.

### Tribological experiments

The friction experiments were conducted using a CSM ball-on-disc tribometer at room temperature, schematically shown in Fig. 1a. The film-coated Si(100) wafer was clamped on a rotating sample platform, meanwhile employing a SUJ2 bearing steel (or Si_3_N_4_) ball of 6 mm in diameter as the counterpart. In this work, four friction pairs were established to evaluate the superlubricity performances of amorphous carbon: a-C:H coated SUJ2 ball vs a-C:H coated Si wafer, bare SUJ2 ball vs a-C:H coated Si wafer, bare Si_3_N_4_ ball vs a-C:H coated Si wafer, and a-C:H:Si coated SUJ2 ball vs a-C:H:Si coated Si wafer. A dry N_2_ environment was produced by purging the inert gas into the chamber of the tribometer. Note that the gas tube was mounted near the sliding contact area to suppress the approaching of the residual gaseous species such as H_2_O and O_2_ in the chamber. The applied normal load could be controlled in the range of 1–10 N, yielding initial Hertz peak contact pressures of 0.5–1.2 GPa. Before each friction test, lateral force calibration for the tribometer load cell should be implemented to ensure the measurement reliability since any zero-shift could result in inaccurate outcomes. The sliding velocity was set at 15–20 cm s^−1^. All the friction experiments were repeated 5–10 times to ensure the reproducibility of the recorded results.

### Characterization of the contact areas

As schematically depicted in Fig. 1b–d, the microscopic probing of the contact areas after friction tests mainly consists of two parts: pre-characterization using various basic techniques (Fig. 1b) and deep-going characterization utilizing scanning electron transmission microscope and electron energy loss spectroscopy (Fig. 1c, d).

The contact areas including the ball wear scars and the wafer wear tracks were imaged using a Nikon optical microscopy. The topographies and sectional profiles of the wear tracks were recorded by Zygo NewView laser-interference surface profilometer. In some cases, the morphologies and surface roughness of the contact areas were characterized by atomic force microscopy (AFM, Asylum Research MFP 3D) using a commercial silicon tip in tapping mode. The bonding structure and configuration, especially the disorder or clustering of C-*sp* ^2^ phase in the carbonaceous tribolayer formed in the contact area, was detected by a visible Raman spectroscopy (Horiba JobinYvon HR800) with Ar^+^ laser wavelength of 514.5 nm. In the case of self-mated a-C:H:Si surfaces, the mechanical properties including the hardness and elastic modulus of the contact areas were evaluated by Nanoindentation system using a Berkovich diamond tip as the indenter.

The lamellar specimens for TEM, STEM and EELS characterization were prepared by a dual-beam scanning electron microscopy (SEM)/focused ion beam (FIB) system (FEI Quanta 3D FEG) based on site- and material-specific lift-out technique. This system is capable of low-kV Ga^+^ ion milling, meanwhile delivering superior SEM images for positioning, as shown in Fig. 1c. To fabricate samples with sufficient quality for atomic-resolution imaging by STEM, we generally adopted the FIB-milling process in ref. ^48^. Before milling, the contact areas were coated by a protective layer of Au or Cr in an ion sputtered system. Then, the samples were transferred into the SEM-FIB chamber. During the whole FIB-milling process, careful adjustments of parameters (i.e., ion beam voltage, beam current, grazing angle, probe size) were carried out to keep the surface protective layers intact, allowing features of interest, especially the area close to the sliding interface, to be analyzed without structural damage or implantation artefacts. Lamellas as thin as 30–60 nm or even lower over large areas up to a dozen micrometers were successfully fabricated, which were qualified for quantitative atomic-resolution STEM and EELS characterization.

For STEM *z*-contrast imaging observation, the state-of-the-art dual-aberration-corrected cold field emission gun STEM (JEOL JEM-ARM200F) was employed. The acceleration voltage was 200 kV, yielding a bright field (BF) imaging resolution of 0.14 nm and a high angle annular dark field (HAADF) resolution of 0.08 nm. The probe convergence semi-angle was 25 mrad. For HAADF image acquisition, the collection semi-angle of 50–180 mrad was used, while BF image was recorded using 11 mrad collection semi-angle. For EELS measurements, the equipped GIF Quantum spectrometer (Gatan) was used. An energy dispersion of 0.25 eV per channel was employed. The probe size was set at 5 C (~ 0.5 nm). The energy resolution, measured at full width of half maximum, was around 1 eV. During recording of core-loss EELS spectra, the DualEELS function in the Gatan Software was used for simultaneous acquirement of one core-loss and one low-loss spectra from an identical sample area. The purpose for doing this was, on one hand, to overcome large energy shift during data acquirement, and on the other hand, to remove plural scattering effect in the core-loss edges when deconvoluted by the low-loss spectrum. Due to the sensitivity of carbon to high-energy electron irradiation, the EELS acquisition time should be set at an appropriate value to avoid any structural transformation and irradiation damage in the specimen. In the present work, the acquisition time for zero-loss and core-loss spectra were set at 0.002 and 0.05–0.1 s per pixel, respectively. By doing this, we had to sacrifice the smoothness of the recorded EELS curves, to some extent. It should be pointed out that, in some cases a conventional field emission high-resolution TEM (HRTEM, JEOL 2010F) operating at 200 kV was first employed for a rough observation before the STEM step.

### Calculation of mass density based on plasmon energy

The recorded EELS spectra usually include zero-loss (0 eV), low-loss (0–50 eV), and core-loss (>50 eV) spectra. The dominating peak in the low-loss spectrum is the plasmon peak ascribed to excitation of all valence electrons. A well-known relationship between the plasmon energy *E*_p_ and the valence electron density *n*_e_ has been established, given by^49^:

1 $$E_{\mathrm{p}} = h\left[ {n_ee^2/m^*{\it{\epsilon }}_0} \right]^{1/2}$$

where *h* is the Planck’s constant, *n*_e_ is the valence electron density, *e* is the electron charge, m* is the effective electron mass and *∈*_0_ is the permittivity of free space. For a multicomponent structure, the average value of *n*_e_ is the summation of its constituent elements weighted by their mole fractions. Therefore, the mass density can be derived as^50,51^:

2 $$\rho _{\mathrm{m}} = \frac{{m_e{\it{\epsilon }}_0E_p^2\mathop {\sum }\nolimits_{k = 1}^n M_kF_k}}{{h^2e^2N_A\mathop {\sum }\nolimits_{k = 1}^n C_kF_k}}$$

where *m*_e_ is the electron mass, *N*_A_ is the Avogadro constant, *M*_*k*_ is the molar mass, *C*_*k*_ is the number of valence electron contributed by atom *k*, *F*_*k*_ is the relative atomic fraction of atom *k*. Note that the effective electron mass m* can be expressed as: m* = χ*m*_e_, where *χ* = 0.87^52^. According to the above assumptions, the measured plasmon energy *E*_p_ of a standard HOPG sample was 26.4 eV, yielding a mass density of 2.16 g cm^−3^ (Fig. 1e, f). Note that during the calculation of a local mass density in the tribolayer, we neglected the contribution of hydrogen since its relative atomic fraction was unknown. Therefore, the calculated density is relatively larger than the actual value.

### Calculation of π* and σ* bonding fractions in EELS C-*K* edge

The low-loss and C-*K* edge spectra are proposed to be used in determining the fractions of carbon atoms hybridized in *sp* ^2^ and *sp* ^3^ configurations. For hydrogenated amorphous carbon materials, this bonding ratio could be calculated from the low-loss spectrum using the Kramers–Kronig analysis method^53^. However, several critical issues limit the accuracy with this method. One is the difficulty in distinguishing the superimposed contribution from π* and σ* transition states. Another one is the effect from multiple scattering. The third one is the contribution from the surface excitations. Therefore, we adopted the approach based on the 1 *s* → π* peak in the C-*K* edge, following Berger et al^54^. In the core-loss spectra, the ionization edges and their near-edge fine structures can be interpreted for analysis of the bonding state, electronic structure and coordination density^55^. For the carbon *K*-edge spectrum (i.e., 280–310 eV measured in an HOPG sample, Fig. 1g), the peak in the range of 285–290 eV (usually centered at ~ 285.5 eV) originates from the excitation of electrons in the ground-state 1 *s* core level to the antibonding π* state (1 *s* → π* transition). Excitation to higher energy level above 290 eV corresponds to the antibonding σ* state (1 *s* → σ* transition). The 1 *s* → π* peak is well identified and the contribution from 1 *s* → π* transition is small in all forms of carbon^56^. To calculate the *sp* ^2^ fractions in C *K*-edge, the π* peak at ~ 285–285.5 eV was fitted using a Gaussian peak, and its area was normalized to the total (π* + σ*) area integrated in the energy window of 280–310 eV. This ratio was then referenced to the standard value obtained for a 100% *sp* ^2^-bonded standard sample (i.e., HOPG in Fig. 1g), finally yielding the *sp* ^2^ fraction in the unknown sample^52,54,57^:

3 $$sp^2{\mathrm{\% }} = \frac{{A_{\mathrm{S}}\left( {\pi ^{\mathrm{*}}} \right)}}{{A_{\mathrm{G}}\left( {\pi ^{\mathrm{*}}} \right)}} \cdot \frac{{A_{\mathrm{G}}\left( {{\mathrm{\Delta }}E} \right)}}{{A_{\mathrm{S}}\left( {{\mathrm{\Delta }}E} \right)}}$$

where *A*_S_(π*), *A*_G_(π*) are the areas of π* peaks for the sample and the standard HOPG, while *A*_S_(ΔE), *A*_G_(ΔE) are the corresponding integrated areas in the energy window of 280–310 eV. In this work, 100% *sp* ^2^-bonded highly oriented pyrolytic graphite (HOPG, ZYA grade, NT-MDT) was used as the EELS standard sample, and the standard value is about 0.12 (Fig. 1g). Note that, due to the highly anisotropic characteristic of HOPG, the EELS measurements were performed approximately at the magic angle condition^52,55,57,58^ by choosing an optimized collection semi-angle (see descriptions in Supplementary Note 3), for removing the preferential orientation effects in EELS spectra. For the 1 *s* → σ* transition, several carbon peaks in *sp* ^3^ bonding configuration have also been well defined, i.e., C–H bond at ~ 287 eV^59^ and C-Si bond at ~ 291.5 eV^60^, which further allow for the identification of their contributing fractions in the C-*K* edge. As discussed below, some oxygen-adsorption-induced bonds such as C–O at ~ 290.8 eV^61^ and C=O at ~ 304 eV^62^ contributing to the σ* transition state were neglected due to their low concentration, for the sake of facilitating the EELS fitting procedure. Note that each EELS spectrum should be background subtracted and then deconvoluted by the Fourier-ratio function using the simultaneously recorded zero-loss spectrum to remove the plural scattering effect. All the data processing was carried out in the Gatan DigitalMicrograph software. However, it should be pointed out that during EELS C-*K* edge quantification, multiple factors such as deconvolution procedure (mainly plural scattering effect), core-hole lifetime broadening effect, variable parameters (energies, width and height) in functional fitting approach as well as the reference standard establishment can affect the quantitative results for each individual carbon bond. Moreover, the less-than-ideal curve smoothness in some C-*K* edge curves due to the relatively short acquisition time (Supplementary Note 1) further impose an obstacle to obtain absolutely accurate quantitative characterization. These varieties and instabilities deteriorate the precision of calculated bond fractions to a noticeable extent, namely with a fluctuation of around 10%. Therefore, based on this accuracy level, we prefer to sort the present EELS analysis results into semi-quantitative or a qualitative assessment (see more details in Supplementary Note 2).

### Influence of oxygen adsorption on structural analysis

After friction test in dry N_2_, it was inevitable to expose the contact area to the ambient atmosphere during transfer of the sample. Hence, oxygen adsorption and structure modification to some extent might occur on the sample surface. This was true when in view of the oxygen signals detected by EDS and EELS in the tribolayers (i.e., Figs. 3e, f, 5j;Supplementary Figs. 5d, 7c,9f, i). This would probably pose an additional obstacle to accurately resolve the nanostructure of the tribolayer. However, we were still capable of distinguishing the adsorbed oxygen from the pristine oxygen existed as the oxidized layer on the steel ball surface. In general, oxygen in the iron oxide could be detected by EELS from the shape of the recorded O-*K* core edge^63^. As shown in Figs. 3f and 5j, an obvious peak split at ~ 530 eV appeared for oxygen in the iron oxide, while only a broad peak at ~ 537 eV was present for other O–*K* edges. To reduce the complexity of fitting procedure, the contribution of oxygen to the C-*K* edge such as C–O and C-= O bonding configuration was neglected, in consideration of the relative low concentration of the adsorbed oxygen in the tribolayer (i.e., Supplementary Figs. 5d and 7c). The same strategy was adopted for other adsorbed trace elements such as nitrogen detected in the tribolayer. On the other side, however, the oxygen signal could be used as an indicator for tracking the structural evolution of the formed tribolayer. For example, the detection of O-*K* signals with a depth up to ~ 30 nm implied the adsorption of oxygen from the environmental atmosphere in the loose and porous tribolayer. This local region actually corresponded to the phase transformation band, which was confirmed by EDS and EELS fitting results (Supplementary Fig. 9i, k).

### Data availability

All the data that support this study are available from the corresponding author upon request.

## Electronic supplementary material

Supplementary Information
