# Supplementary material for: Evolution of tribo-induced interfacial nanostructures governing superlubricity in a-C:H and a-C:H:Si films
Source: Nat Commun. 2017 Nov 22;8:1675. doi: 10.1038/s41467-017-01717-8 (PMC5698435; doi:10.1038/s41467-017-01717-8)
Supplement: Supplementary file 1 — Supplementary Information [file 41467_2017_1717_MOESM1_ESM.pdf]

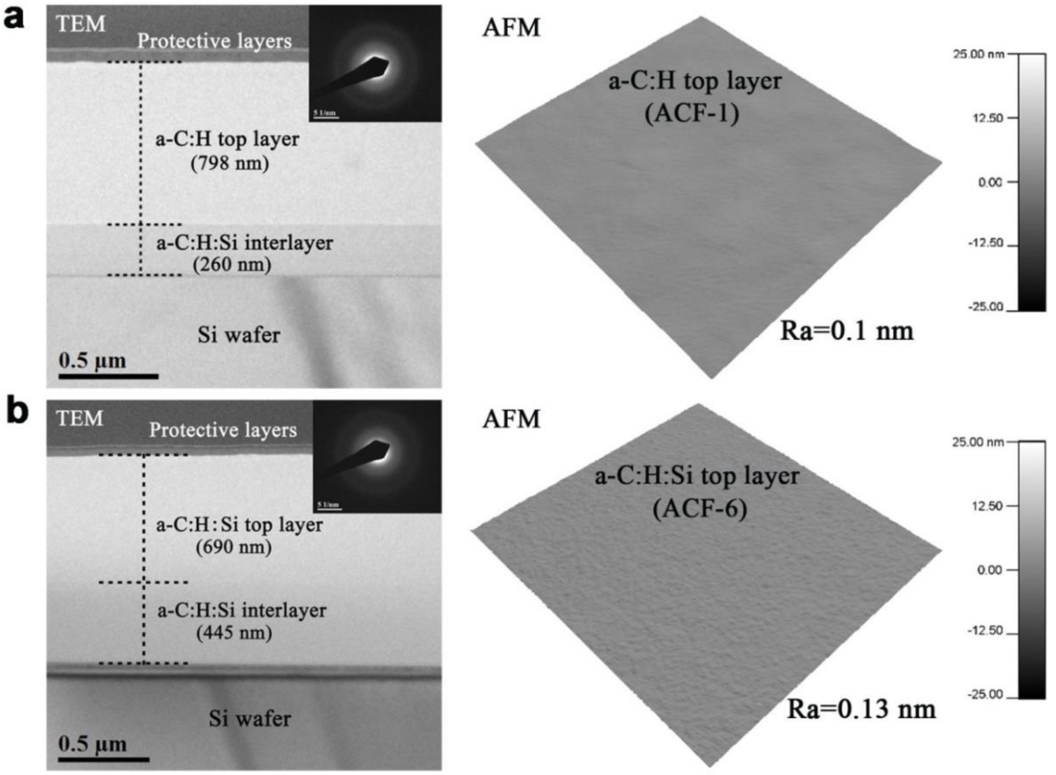

c

Code	Grown films	Composition (at.%)					Hardness (GPa)	Elastic modulus (GPa)	Roughness (nm)
		H	C	Si	O	N			
ACF-0	a-C:H:Si interlayer	17.5	70.7	8.9	2.1	0.8	23.3	232.6	0.17
ACF-1	a-C:H top layer	39.3	59.4	0.2	0.1	1.0	13.4	112.5	0.10
ACF-6	a-C:H:Si top layer	31.9	57.2	9.3	1.2	0.4	16.2	155.7	0.13

Supplementary Figure 1 | Nanostructures and properties of the as-grown hydrogen-rich a-C:H (ACF-1) and a-C:H:Si (ACF-6) films on Si wafers. A hydrogen-deficient and hard a-C:H:Si interlayer was employed to act as a bonding layer for the both films. (**a**,**b**) Low-magnification TEM images (left side) showing the bilayer structure of the as-grown films. (Insets) Electron diffraction pattern images demonstrating the typical amorphous characters of the as-grown a-C:H (ACF-1) and a-C:H:Si (ACF-6) films. AFM three-dimensional morphological images (right side, 10 μm × 10 μm) indicating the atomically smooth surfaces of the both films. The detailed growth procedure and parameters can be found elsewhere (refs 1, 2). (**c**) Compositional and mechanical properties of the as-grown individual layers. ACF denotes ‘amorphous carbon film’. The elemental composition was measured by ERDA and XPS. The hardness and elastic modulus was determined by nanoindentation. The surface roughness was recorded by AFM.

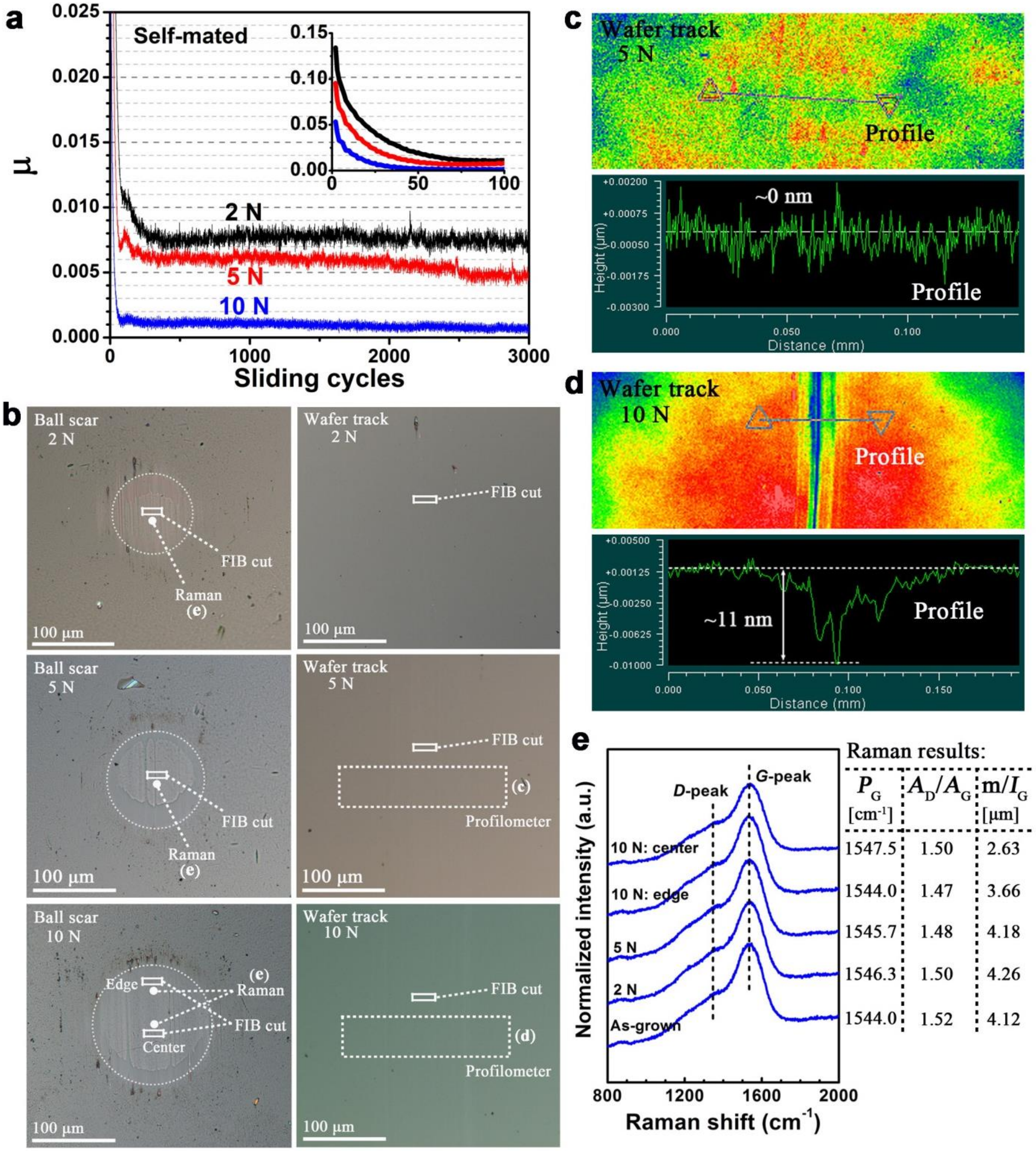

Supplementary Figure 2 | Superlubricity behaviors and basic characterizations of the contact areas for the self-mated a-C:H (ACF-1) surfaces sliding at various normal loads. (**a**) Friction coefficients μ quickly evolving to steady-state values of 0.008, 0.0055 and 0.001 at normal loads of 2, 5 and 10 N, respectively, at the onset of sliding contact in dry N_2 atmosphere. The initial peak (average) Hertz contact pressures at loads of 2, 5 and 10 N were calculated to be 0.68 (0.46), 0.93 (0.62) and 1.17 (0.78) GPa, respectively. The sliding speed was 15 $cm{\cdot}s^{-1}$. The inset shows the zoomed first 100 sliding cycles, in which a shorter running-in stage and a lower initial μ are observed at a higher contact pressure. (**b**) Optical images showing the wear scars and the wear tracks (barely visible to naked eyes) produced on the ball and wafer surfaces. The diameters of the wear scars were measured to be 97.7, 118.5 and 143.6 μm at normal loads of 2, 5 and 10 N, respectively. The corresponding steady-state apparent average contact pressures were calculated to be 0.27, 0.45 and 0.62 GPa,

respectively. It seems that the self-mated hydrocarbon surfaces under a higher normal load (i.e., 10 N) were still rubbing at a higher contact pressure in the steady state even though the contact area is substantially enlarged as compared with the cases of lower normal loads (i.e., 2 and 5 N). According to the equation $\mu=S_0/P$ (ref. 3) for solid lubricants, we can roughly estimate the interfacial shear strength S_0 in the steady state. The calculated values were 2.16, 2.47 and 0.62 MPa, respectively, for normal loads of 2, 5 and 10 N. There was a noticeable reduction of S_0 for the load of 10 N, implying a pressure-induced change of the bonding structure of the sliding interface (see Fig. 2e,h). (**c**,**d**) Interference images showing the sectional profiles across the wafer wear tracks generated at loads of 5 and 10 N, as marked in **b**. Wear depths of ~0 and ~11 nm were recorded for the both a-C:H wear tracks, respectively. (**e**) Comparison of Raman spectra (left side) measured from the as-grown a-C:H film, the ball wear scar center at 2 N, the ball wear scar center at 5 N, the ball wear scar edge and the scar center at 10 N, as marked in **b**. The fitting results corresponding to each curve are presented simultaneously (right side). Based on Raman theory for amorphous carbon (refs 4, 5), the almost invariable *G*-peak position P_G (~1545 cm^{-1}) and peak area ratio A_D/A_G (~1.5) illustrate the nearly unaffected bonding structures of a-C:H layers in the contact areas. Note that Raman signal could probe the subsurface deep to several hundred nanometers, therefore the detected information mainly reflected the properties of the bulk. However, the decreased photoluminescence background m/I_G at 10 N, a measure index for hydrogen content in the film (ref. 4), implies the reduction of the detected signal volume of hydrogen atoms. This is expected to probably originate from the bulk lateral extending of the film layers (Supplementary Fig. 3) and possible hydrogen release from the film surface upon sliding contact.

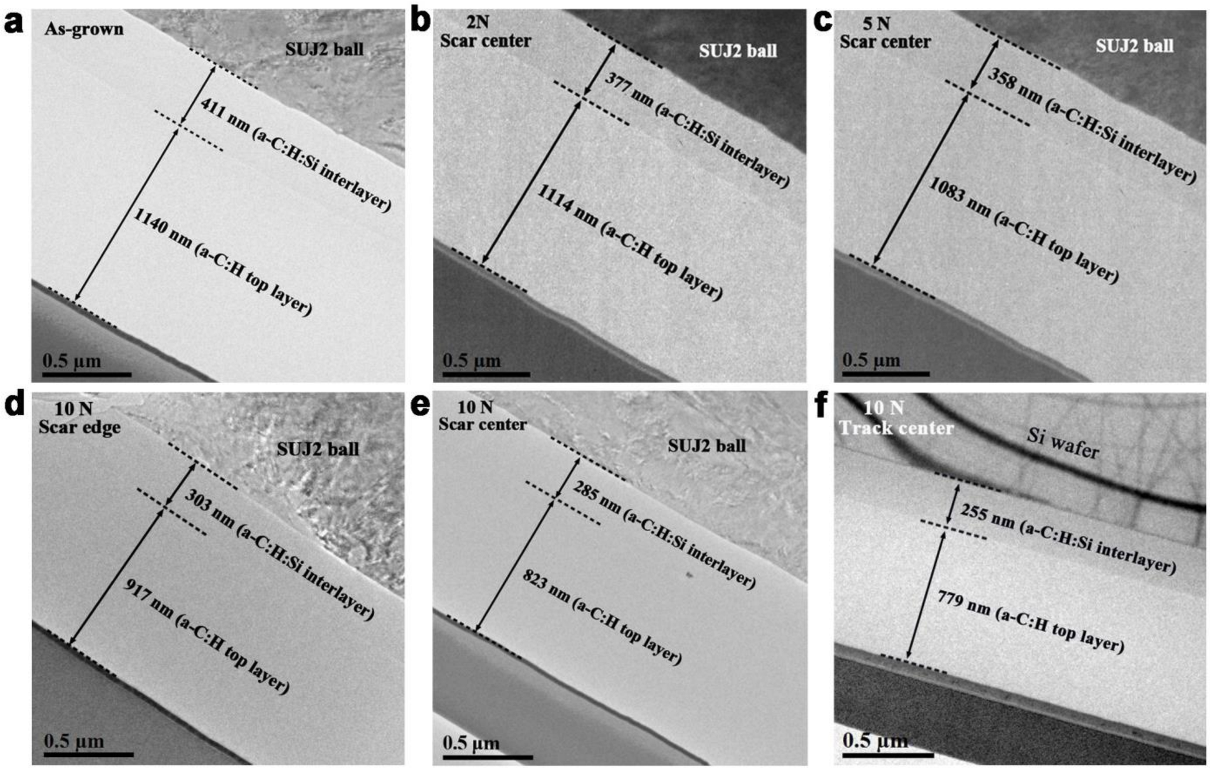

Supplementary Figure 3 | TEM characterization of the contact areas for the self-mated a-C:H (ACF-1) surfaces after the superlubricity tests shown in Supplementary Fig. 2. (**a**–**f**) Low-magnification TEM images showing the cross-sectional structures of the contact areas at various loads: (**a**) the as-grown a-C:H film on SUJ2 steel ball, (**b**) ball wear scar center at 2 N, (**c**) ball wear scar center at 5 N, (**d**) ball wear scar edge at 10 N, (**e**) ball wear scar center at 10 N and (**f**) wafer wear track center at 10 N. The thicknesses of the a-C:H:Si interlayer and the a-C:H top layer gradually decreased with the increase in load.

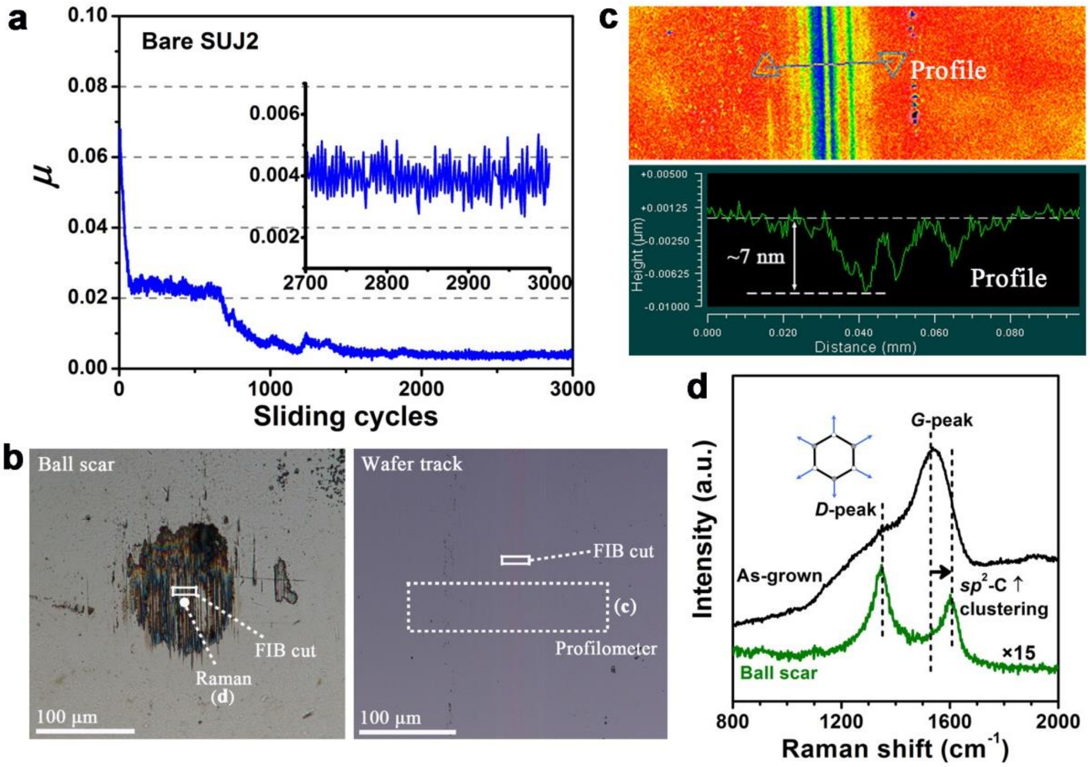

Supplementary Figure 4 | Superlubricity behavior and basic characterization of the contact areas for the friction pair of bare SUJ2 steel ball and a-C:H (ACF-1) surface. (**a**) Friction coefficient μ reaching a steady-state value of 0.004 after a running-in period (μ~0.025) in dry N_2 atmosphere. Tribotesting condition: normal load of 2 N (initial peak Hertz contact pressure of 0.68 GPa) and sliding speed of 15 $cm \cdot s^{-1}$. (**b**) Optical images showing the wear scar and the wear track (barely visible to naked eyes) produced on the ball and wafer surfaces. (**c**) Interference images presenting the sectional profile across the wafer wear track as marked in **b**. A wear depth of ~7 nm was detected in the a-C:H wear track. (**d**) Comparison of Raman spectra measured from the as-grown a-C:H film and the ball scar center as marked in **b**. The split and sharpening of *D*-peak along with the up-shift of the *G*-peak position implying the remarkable increase and local clustering of sp^2-C phase (ref. 5) in the tribolayer produced on the ball wear scar surface.

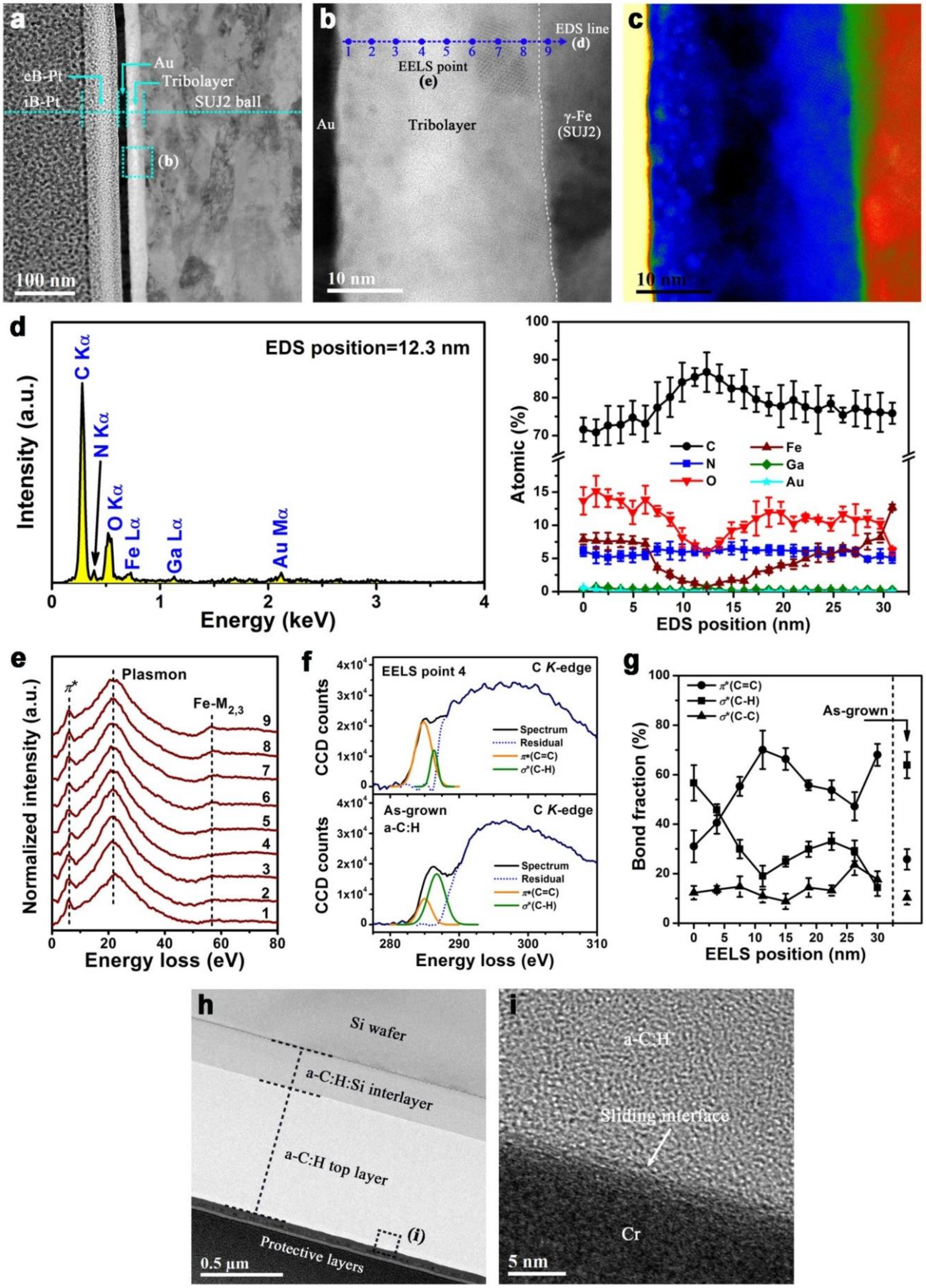

Supplementary Figure 5 | TEM, STEM and EELS characterization of the contact areas for the friction pair of bare SUJ2 steel ball and a-C:H (ACF-1) surface after the superlubricity test shown in Supplementary Fig. 4. (**a**) Low-magnification TEM image showing the formation of a nanometer-thick

carbonaceous tribolayer on the SUJ2 ball scar surface. (**b**) BF-STEM image presenting the enlarged details of the tribolayer as marked in **a**. (**c**) False-color displayed HAADF image corresponding to **b**. (**d**) A typical EDS spectrum and the evolution of elemental composition across the tribolayer as marked in **b**. *Error bars* represent s.d. of the measured atomic composition. (**e**) Evolution of low-loss spectrum across the tribolayer as marked in **b**. (**f**) Examples of peak resolving to the C *K*-edges measured from the as-grown a-C:H film and the EELS point 4 in the tribolayer as marked in **b**. Two Gaussian peaks are fitted to π*(C=C) and σ*(C-H) bonds, respectively. The residual bond fraction for C-hybridization is then assigned to σ*(C-C), neglecting the contribution from C-O and C=O bonds. (**g**) Evolution of the calculated EELS C-bonds fractions across the tribolayer as marked in **b**. *Error bars* denote s.d. of calculated bond fractions. (**h**) Low-magnification TEM image showing the cross-sectional structure at the center of the wafer wear track. (**i**) HRTEM image showing the atomic structure of the sliding interface as marked in **h**. The bonding structure of the a-C:H top layer along the sliding interface is nearly intact as compared to the as-grown a-C:H film (see Supplementary Fig. 1a).

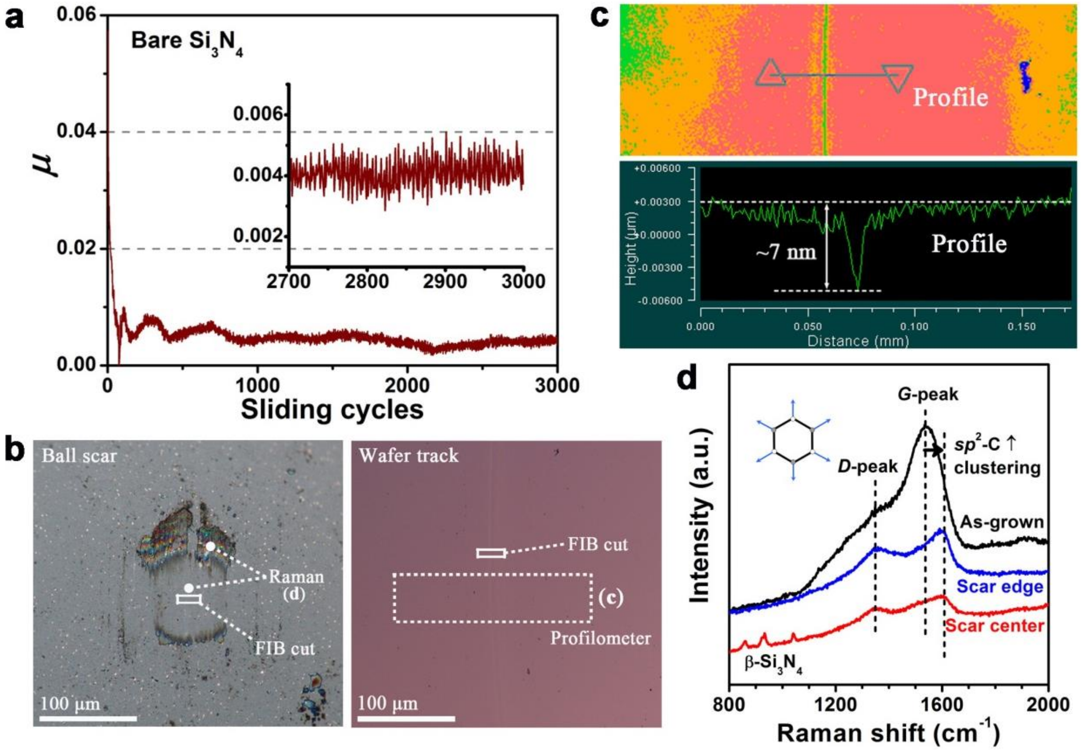

Supplementary Figure 6 | Superlubricity behavior and basic characterization of the contact areas for the friction pair of bare Si_3N_4 ball and a-C:H (ACF-1) surface. (**a**) Friction coefficient μ quickly reaching a steady-state value of 0.004 at the onset of sliding contact in dry N_2 atmosphere. Tribotesting condition: normal load of 2 N (initial peak Hertz contact pressure of 0.75 GPa) and sliding speed of 15 $cm \cdot s^{-1}$. (**b**) Optical images showing the wear scar and the wear track (barely visible to naked eyes) produced on the ball and wafer surfaces, respectively. (**c**) Interference images showing the sectional profile across the wafer wear track as marked in **b**. A wear depth of ~7 nm was detected in the a-C:H wear track. (**d**) Comparison of Raman spectra measured from the as-grown a-C:H film, the ball wear scar edge and the ball wear scar center as marked in **b**. The split and sharpening of *D*-peak along with the up-shift of the *G*-peak position suggesting the remarkable increase and local clustering of sp^2-C phase (ref. 5) in the tribolayer generated on the ball wear scar surface. The detection of Raman signal from the underlying Si_3N_4 (850-1050 cm^{-1}) at the ball scar center implies that the thickness of the tribolayer is in nanometer scale.

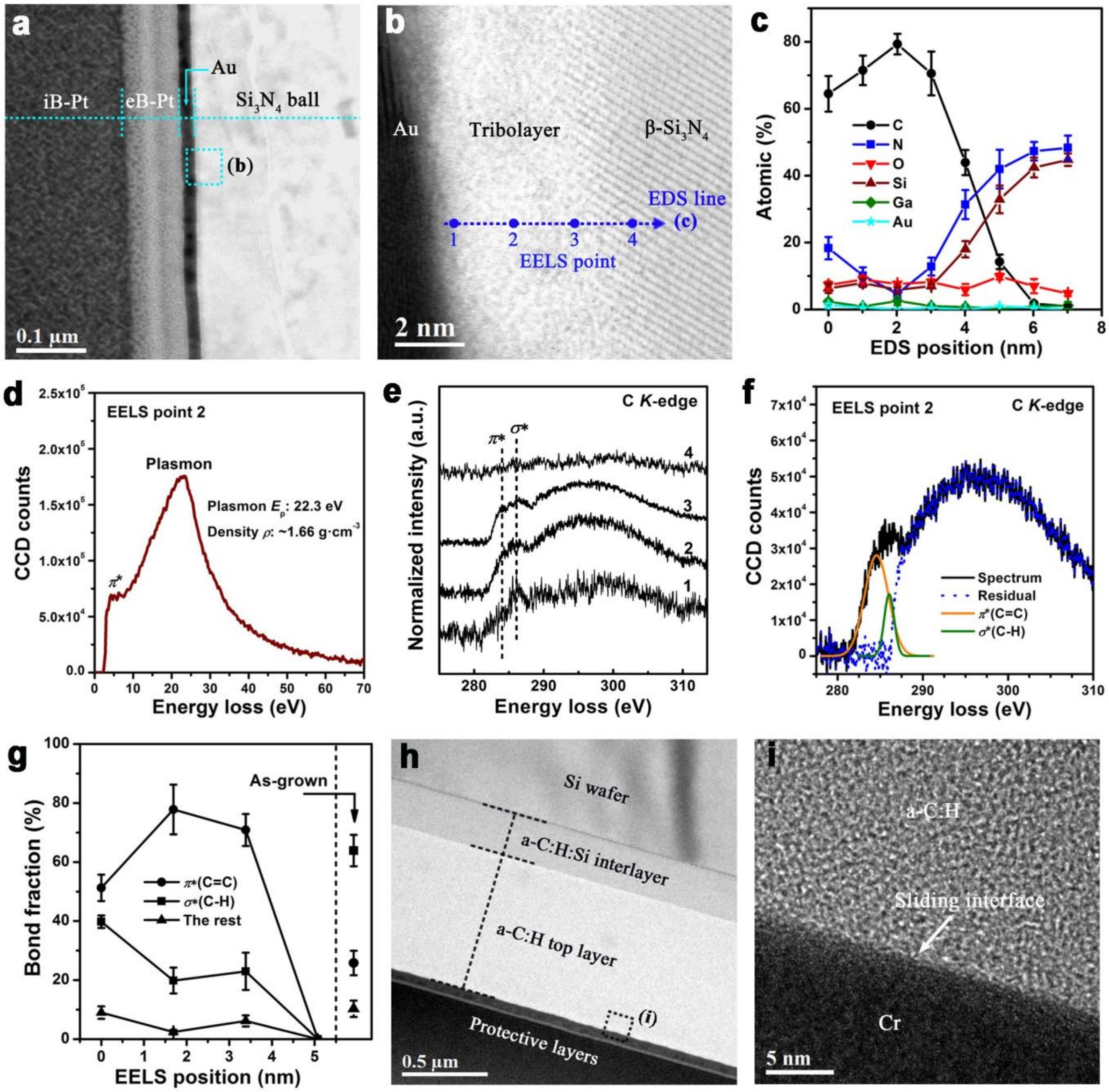

Supplementary Figure 7 | TEM, STEM and EELS characterization of the contact areas for the friction pair of bare Si_3N_4 ball and a-C:H (ACF-1) surface after the superlubricity test shown in Supplementary Fig. 6. (**a**) Low-magnification TEM image showing the cross-sectional morphology of the Si_3N_4 ball wear scar. (**b**) STEM-BF image presenting the enlarged details of the tribolayer formed on the Si_3N_4 surface as marked in **a**. (**c**) Evolution of the elemental composition across the tribolayer as marked in **b**. *Error bars* represent s.d. of the measured atomic composition. (**d**) Deconvoluted low-loss spectrum from the EELS point 2 in the tribolayer as marked in **b**. The measured plasmon energy E_p of 22.3 eV yielding a mass density of about 1.66 g·cm^{-3}. (**e**) Evolution of C *K*-edge across the tribolayer as marked in **b**. (**f**) Peak resolving to the C *K*-edge measured from the EELS point 2 in the tribolayer as marked in **b**. Two Gaussian peaks were fitted to π*(C=C) and σ*(C-H) bonds, respectively. The residual fraction for C-hybridization is the total amount of C-C, C-O, C=O and other possible bonds. (**g**) Evolution of the calculated EELS C-bonds fractions across the tribolayer as marked in **b**. *Error bars* define s.d. of calculated bond fractions. (**h**) Low-magnification TEM image showing the cross-sectional structure at the center of the wafer wear track. (**i**) HRTEM image showing the atomic structure of the sliding interface as marked in **h**. The bonding structure of the a-C:H top layer along the sliding interface is nearly intact as compared to the as-grown a-C:H film (see Supplementary Fig. 1a).

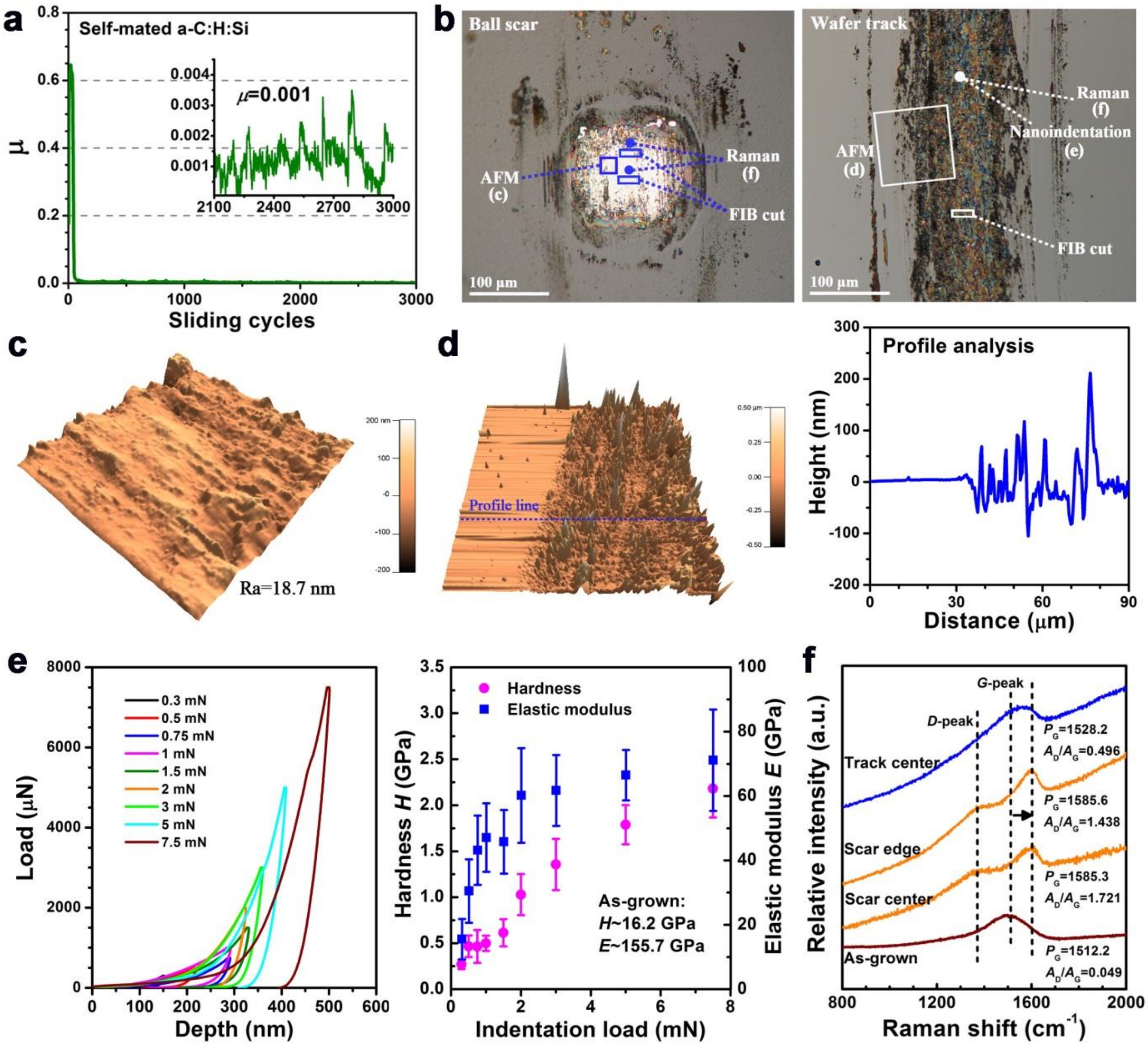

Supplementary Figure 8 | Superlubricity behavior and basic characterization of the contact areas for the self-mated a-C:H:Si (9.3 at.% Si, ACF-6) surfaces. (**a**) Friction coefficient μ quickly reaching a steady-state value of 0.001 at the onset of sliding contact in dry N_2 atmosphere. Tribotesting condition: normal load of 2 N (initial Hertz contact pressure of 0.68 GPa) and sliding speed of 20 $cm \cdot s^{-1}$. (**b**) Optical images showing the wear scar and the wear track produced on the ball and wafer surfaces. A thick tribolayer was generated in situ covering the wafer wear track due to the material transfer from the ball surface to the wafer side. **a** and **b** reproduced with permission from ref. 2. Copyright 2014 American Chemical Society. (**c**) AFM-3D (10 μm × 10 μm) image showing the smooth wear scar surface with roughness *R*a=18.7 nm, as marked in **b**. (**d**) AFM-3D (90 μm × 90 μm) and sectional profile images indicating the rough morphology of the tribolayer in the wear track, as marked in **b**. The average thickness was in the range of 50-100 nm. (**e**) Load-dependent nanoindentation measurements revealing the flexibility and softness of the formed tribolayer. The intrinsic hardness is about 0.25 GPa measured at the small load of 0.3 mN (avoiding the effect from the underlying layer and substrate), quite lower than the as-grown a-C:H:Si film (16.2 GPa). *Error bars* represent s.d. of the measured values. (**f**) Comparison of Raman spectra measured from the as-grown a-C:H:Si film and the contact areas. The increase in A_D/A_G ratio and the up-shift of *G*-peak position demonstrating the enhancement of sp^2-phase present in the contact areas.

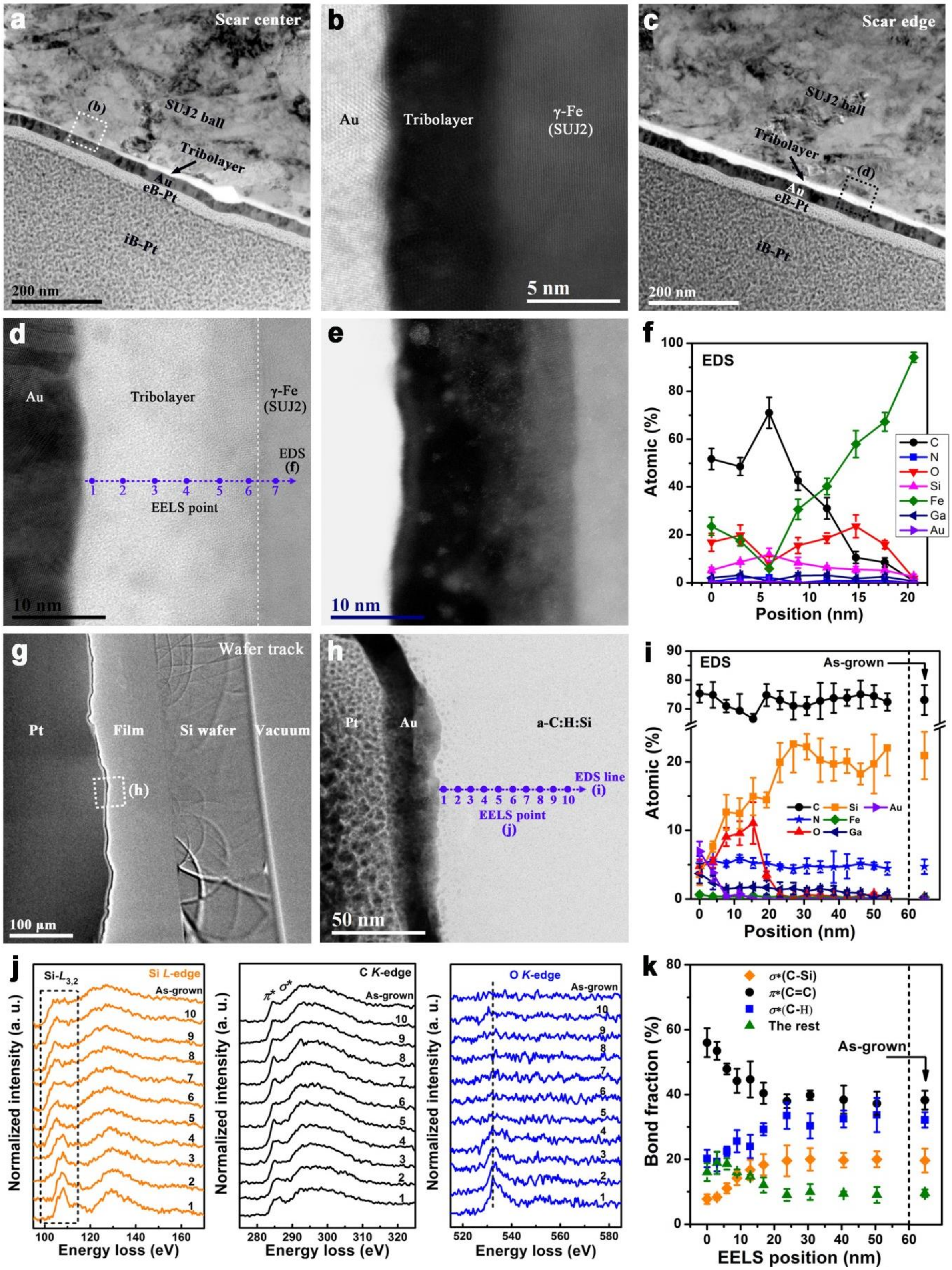

Supplementary Figure 9 | TEM, STEM and EELS characterization of the contact areas for the self-mated a-C:H:Si (9.3 at.% Si, ACF-6) surfaces after the superlubricity test shown in Supplementary Fig. 8. (**a**) Low-magnification TEM image showing the cross-sectional morphology of the ball wear scar center,

as marked in Supplementary Fig. 8b. (**b**) HAADF-STEM image showing the enlarged detail of the tribolayer (~5 nm) formed on the scar center surface, as marked in **a**. This figure corresponds to the BF-STEM image in Fig. 5a. (**c**) Low-magnification TEM image showing the cross-sectional morphology of the ball wear scar edge, as marked in Supplementary Fig. 8b. (**d**) BF-STEM image showing the enlarged detail of the tribolayer (~20 nm) formed on the scar edge surface, as marked in **c**. (**e**) HAADF-STEM image corresponding to **d**. (**f**) Evolution of the EDS-measured elemental composition across the tribolayer as marked in **d**. *Error bars* represent s.d. of the measured atomic composition. (**g**) Low-magnification TEM image showing the cross-sectional morphology of the wafer wear track, as marked in Supplementary Fig. 8b. Wavy profiles were observed along the sliding interface, confirming the rough morphology of the formed tribolayer as indicated in Supplementary Fig. 8d. (**h**) BF-STEM image showing the enlarged detail of the tribolayer formed in the wear track, as marked in **g**. Note that almost no obvious phase contrast can be distinguished between the tribolayer and the underlying a-C:H:Si layer, mainly due to the same compositional elements and close contents in them. (**i**) Evolution of the EDS-measured elemental composition of the tribolayer from the sliding interface towards the underlying intact a-C:H:Si film, as marked in **h**. Note that a small number of Au and Ga nanoparticles were inevitably implanted into the outer-most region (~10 nm) due to the rough and porous morphology of the formed tribolayer. *Error bars* represent s.d. of the measured atomic composition. (**j**) Evolution of Si-*L*, C-*K* and O-*K* EELS edges recorded across the tribolayer point by point as marked in **h**. All the spectra are displayed after the background subtraction and the followed deconvolution with a corresponding low loss spectrum. (**k**) Evolution of the calculated EELS C-bonds fractions across the tribolayer from the EELS C-*K* edges presented in **j**. *Error bars* denote s.d. of calculated bond fractions.

a

Code	Grown films*	Composition (at.%)					Hardness (GPa)	Elastic modulus (GPa)	Roughness (nm)
		H	C	Si	O	N			
ACF-1	a-C:H layer	39.3	59.4	0.2	0.1	1.0	13.4	112.5	0.10
ACF-2	a-C:H:Si layer	43.5	50.6	4.2	1.1	0.6	11.5	93.6	0.17
ACF-3	a-C:H:Si layer	30.0	63.7	4.9	1.4	0.0	15.6	144.9	0.09
ACF-4	a-C:H:Si layer	35.3	56.3	5.7	1.5	1.2	14.0	123.8	0.14
ACF-5	a-C:H:Si layer	31.1	59.7	8.5	0.2	0.6	16.5	154.0	0.16
ACF-6	a-C:H:Si layer	31.9	57.2	9.3	1.2	0.4	16.2	155.7	0.13

*For all the grown films, a hard a-C:H:Si interlayer (ACF-0, Supplementary Fig. 1c) was first deposited on the substrates to act as a bonding layer. ACF-1 and ACF-6 were the same samples as shown in Supplementary Fig. 1.

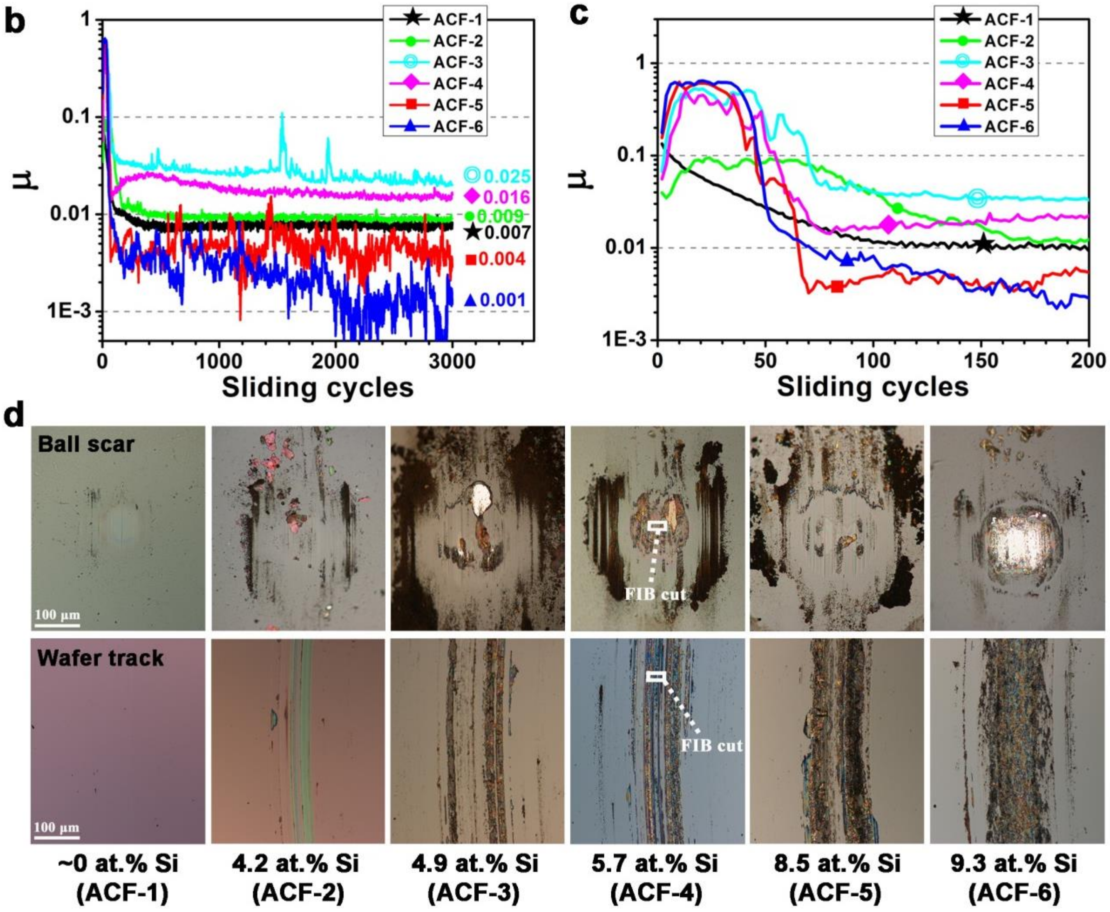

Supplementary Figure 10 | Composition and properties of the as-grown hydrogen-rich a-C:H:Si films with different Si contents and their anti-friction behaviors. (**a**) Atomic composition and mechanical properties of the as-grown hydrogen-rich a-C:H:Si films. The silicon content gradually increases from ~0 at.% to 9.3 at.% in the films of ACF-1 to ACF-6. (**b**) Superlubricity behaviors in dry N_2 atmosphere. Tribotesting condition: normal load of 2 N and sliding speed of 20 $cm{\cdot}s^{-1}$. (**c**) Zoom of the first 200 sliding cycles to highlight the running-in stages. (**d**) Optical images showing the evolution of morphologies of the wear scars and the wear tracks produced on the ball and wafer surfaces. Close relationship could be found between the tribolayer coverage and the Si content, namely, from the absence of tribolayer (~0 at.% Si) to the development of an incomplete tribolayer (4.2-8.5 at.% Si), then to the formation of a fully complete tribolayer (9.3 at.% Si).

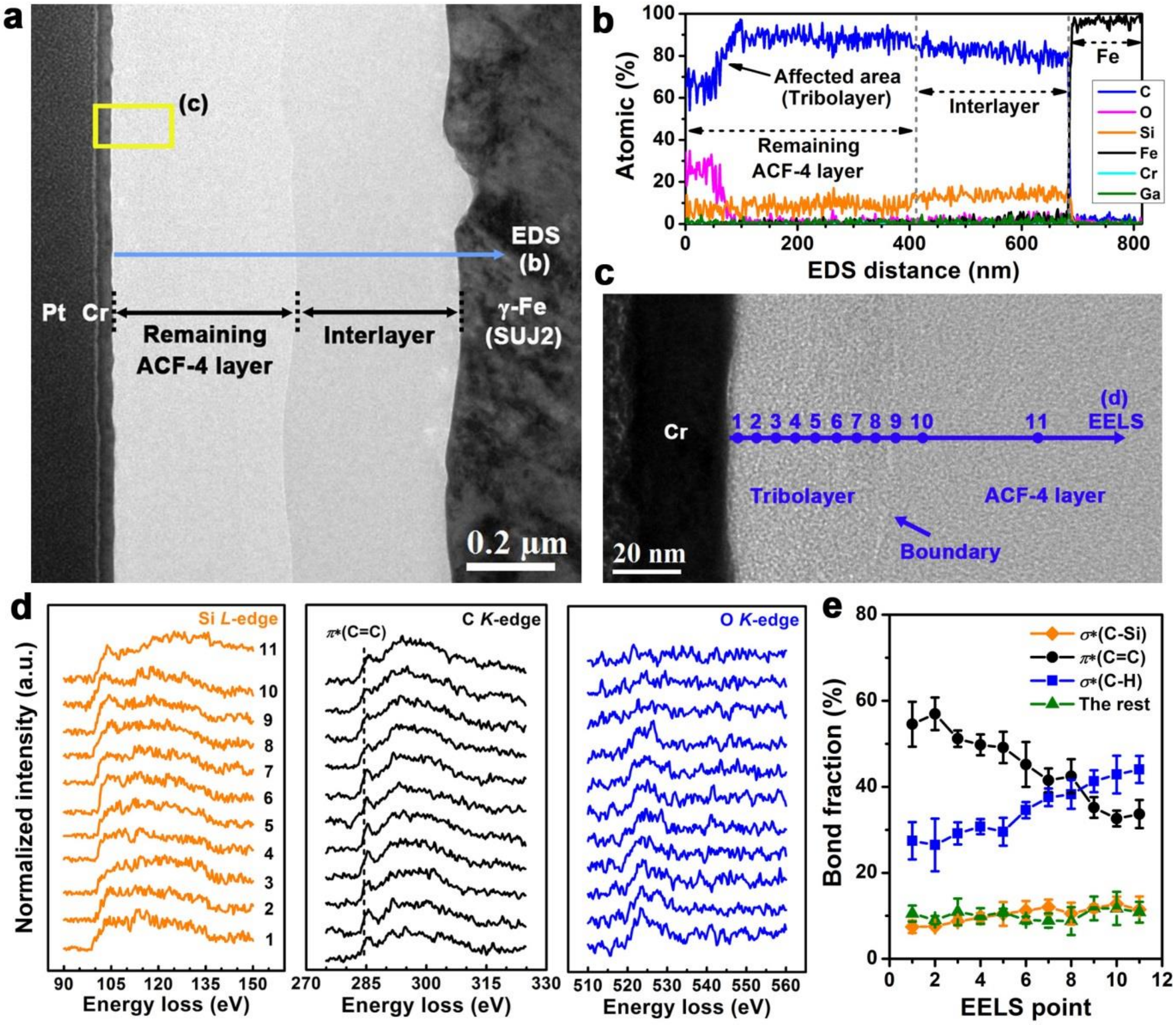

Supplementary Figure 11 | STEM and EELS characterization of the contact area produced on film-coated SUJ2 steel ball surface for self-mated a-C:H:Si (5.7 at.% Si, ACF-4) surfaces after the friction test (Supplementary Fig. 10b). (**a**) Low-magnification TEM image showing the cross-sectional morphology of FIB lamella sliced from the ball wear scar, as marked in Supplementary Fig. 10d. It could be seen that there still remained a part of ACF-4 layer after the material transfer to the wafer side for growth of an anti-friction tribolayer (Supplementary Fig. 10d). (**b**) EDS-elemental distribution across the ball wear scar as marked in **a**. An outermost area in the remaining ACF-4 layer was found to be affected after the friction test when in view of the carbon and oxygen content. The increase of oxygen content was due to the oxygen adsorption from the ambient atmosphere by the nanoporous tribolayer. (**c**) BF-STEM image showing the local morphology of the tribolayer formed in the outermost area of the remaining ACF-4 layer. (**d**) Evolution of Si-*L*, C-*K* and O-*K* EELS core-edge spectra recorded across the tribolayer-covered area point by point as marked in **c**. (**e**) Evolution of the calculated EELS C-bonds fractions from the EELS C-K edges presented in **d**. *Error bars* denote s.d. of calculated bond fractions.

Supplementary Note 1 | The strategy for EELS measurement at 200 kV

In some cases, carbon-based materials are very sensitive to high-energy electron irradiation-induced damage. The degree of damage is highly dependent on the material structure and the incident electron beam[6]. For instance, carbon nanotubes and graphene are usually suffered from knock-on damage[7], namely displacing carbon atoms in the graphene structure. Therefore, it would be best for the characterization of these atomic-scale thin carbon materials using electron microscopes conducted at low acceleration voltages, such as a threshold of about 80 kV for SWNT. For amorphous carbon, the motivation that we still choose the beam voltage of 200 kV for STEM and EELS acquisition is based on the following considerations. Firstly, the dual-aberration-corrected JEOL JEM-ARM200F STEM used in this work is designed to be a 200 kV microscope, which is capable of delivering a STEM-HAADF spatial resolution of 0.08 nm and an EELS energy resolution of 0.34 eV at 200 kV. This powerful sub-angstrom imaging capability is the core requirement for this study, namely resolving the tribo-induced interfacial nanostructures at the atomic scale. Although this microscope can also be operated at 80 kV, the STEM imaging resolution as well as the energy resolution would be reduced to a remarkable extent at this low voltage. This weakened imaging capability thus cannot guarantee the quality of the received images as good as those obtained in this work, which may fail in capturing the atomic details of the tribolayer nanostructures. Secondly, amorphous carbon is more endurable to electron-beam irradiation due to its bulk-like characteristic as compared to the two-dimensional (very thin) structure of graphene. Therefore, EELS spectra can be acquired at higher beam voltage such as 200 kV, by optimizing acquisition time (0.05-0.1 s) to largely suppress structural transformation and irradiation damage in amorphous matrix. Moreover, as shown in Supplementary Fig. 12, the shape of core-loss C-*K* spectrum recorded at 200 kV was almost the same as that of 80 kV. This indicates that similar EELS results could be achieved for both voltages. However, the relatively short acquisition time (0.05-0.1 s) leaded to the reduced smoothness of the acquired EELS spectra or even noisiness in some cases (notably those in Fig. 3f, Fig. 5j and Supplementary Fig. 11d) where carbon content in the tribolayer was not abundant. Under such circumstances, we had to sacrifice, to some extent, the precision of bond fractions obtained through peak fitting. In spite of this, qualitative or semi-quantitative analysis of the interfacial nanostructures based on EELS spectra is definitely possible and reliable when a

properly converged and stable fitting procedure (Supplementary Note 2) is consistently used for the whole set of data, even if the absolute values can be different in some degree.

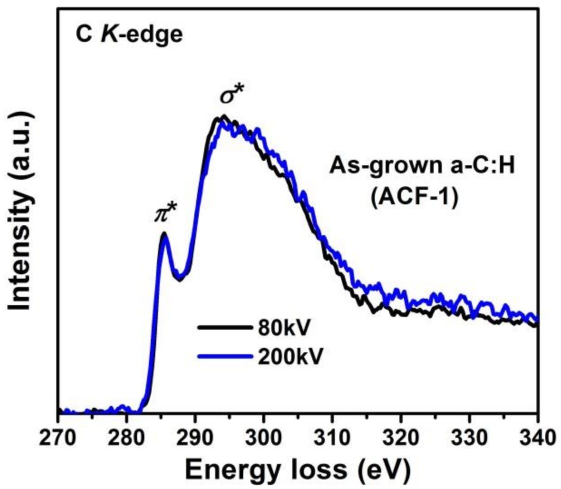

Supplementary Figure 12 | Comparison of EELS carbon *K*-edge spectra recorded at 80 and 200 kV under a relatively short acquisition time of 0.05 s. Note that, for the purpose of clarity, the two spectra are normalized to the same intensity by referring to the peak intensity of C-*K* edge. Obviously, the almost overlapped C-*K* edge curves indicate that similar EELS results could be achieved for both voltages.

Supplementary Note 2 | Calculation technique for EELS C-*K* quantification and the accuracy of the derived π^* and σ^* bond fractions

The high-energy electrons can cause the electrons to excite from 1s core level to unoccupied states, and the energy-loss near edge structure (ELNES) is capable of reflecting the bonding features in C-*K* EELS spectra, namely π^* and σ^* electronic states for carbon atoms. Therefore, the C-*K* edge spectra can be regarded as the superposition of integrated intensities from several core-exciton π^* and σ^* peaks. To determine the individual bond fraction, the key requirement is to distinguish and resolve the corresponding π^* and σ^* peaks from the C-*K* edge, based on the consideration that the number of recorded counts is proportional to the number of π-/σ-bonded electrons. The fractions of sp^2-/sp^3-bonded carbon atoms are derived by referencing the ratio (R) of individual integrated area in the specific energy window to that of standard samples. To extract the ratios of peaks from experimental EELS spectra, a number of Gaussian or Lorentzian functions[8,9] as well as a particular energy window can be used during peak fitting. Various sets of fitting parameters are proposed in different methods[10], and the choice of these parameters is ultimately a decisive factor in affecting the calculated bonds fractions. Numerous efforts in the

literatures are devoted in determining the optimum combinations of these fitting parameters to achieve the smallest fluctuations of the derived sp^2 bond fractions, namely improving the precision of quantification. In this work, we follow a straightforward calculation method well established by Berger and co-workers[11] and other researchers[12,13]. In detail, following L. Ponsonnet et al.[14] and A. J. Papworth et al.[9] (multiple-functional fitting approach), a few Gaussian peaks with specific energies, widths and heights are fitted to the π^* and σ^* features. Note that the π^* and σ^* peaks involved in the present work are as follows: π^*(C=C) at 285.5 eV, σ^*(C-H) at 287 eV, σ^*(C-Si) at 291.5 eV and σ^*(C-C) at 292.5 eV. The R-ratio for each individual peak such as π^* peak was achieved by normalizing its integrated area to the total ($\pi^*+\sigma^*$) area integrated in the energy window of 280-310 eV. This ratio was then referenced to the standard value obtained for a 100% sp^2-bonded sample such as HOPG, yielding the desired bonds fractions in the unknown carbon materials. To largely suppress the fluctuations in the fitting process, improvement could be obtained by constraining some fit parameters (e.g. the energy position of Gaussian peak)[10]. Therefore, during fitting in this work, the energies of each individual peak were fixed, while the widths and heights were variable parameters. However, it should be pointed out that, the peak energies were allowed to adjust manually in a small range around the nominal value by taking small chemical shift into account. Meanwhile, to reduce the complexity and instability during fitting, the deconvolution procedure was relatively simplified by reducing one Gaussian function, namely the σ^*(C-C) peak. Correspondingly, the bond fraction of σ^*(C-C) was achieved by calculating the balance from the total 100%. With fixed peak energies, the Gatan DigitalMicrograph software automatically optimized all these parameters until the deconvolution curve coincided with the experimental spectrum. For instance, during fitting of C-*K* edge of carbonaceous tribolayer formed from a-C:H film, two Gaussian peaks including π^*(C=C) at 285.5 eV and σ^*(C-H) at 287 eV were fitted to the experimental spectrum (Supplementary Fig. 5f). The two energies of 285.5 and 287 eV were adjusted slightly, i.e., within a range of ±0.3 eV, until a good agreement between the sum of fitting peaks and the experimental spectrum was realized.

The calculation accuracy or fluctuation is the key concern in sp^2/sp^3 carbon fractions determination from EELS C-*K* edge. Multiple factors such as deconvolution procedure (mainly plural scattering effect)[15], core-hole lifetime broadening effect[16], variable parameters (energies, width and height) in functional fitting approach[10] as well as the reference standard establishment

(see Supplementary Note 3) can affect the quantitative results for each individual carbon bond. Nevertheless, after suitable technical treatments and careful adjustments of fitting parameters, some researchers could achieve high accuracy by reducing the fluctuations in the EELS calculated bond fractions down to around 2%. In the present work, however, it should be pointed out that in addition to the above spectral issues, the less-than-ideal curve smoothness in some C-*K* edge curves (Fig. 3f, Fig. 5j and Supplementary Fig. 11d) due to the relatively short acquisition time (Supplementary Note 1) further impose an obstacle to obtain absolutely accurate quantitative characterization, even though we have carefully treated all these concerns by taking the well-documented fitting criterions into account. These varieties and instabilities deteriorate the precision of calculated bond fractions to a noticeable extent, namely with a fluctuation of around 10%. Therefore, based on this accuracy level, we prefer to sort the present EELS analysis results into semi-quantitative or a qualitative assessment. As mentioned above, a quantitative processing of the whole set of spectra consistently following a properly converged and stable fitting procedure at least allows a reliable qualitative analysis of the structural evolution in the targeted carbon materials.

Supplementary Note 3 | Suppression of orientation effect on EELS core edges under 'magic angle' measurement condition

As well recognized, ELNES measurements in crystalline materials are sensitive to orientation of the ordered local structure, namely the effect of anisotropy[17]. Even though the crystal orientation dependence is not the concern in amorphous carbon, the standard reference sample of HOPG used in this work would be affected by this anisotropic effect[9]. Theoretical studies have shown that there exist experimental conditions where the anisotropic effect can be cancelled when a particular collection semi-angle (β) is used[18]. This angle is the so-called magic angle. Values calculated from theoretical models for the magic angle could vary from 1.36 θ_E to 4θ_E[19,20], where θ_E was the energy-dependent characteristic scattering semi-angle of electrons. However, in practical application, there was big discrepancy between the experimental value and the theoretical one, which was speculated to be due to various possibilities such as contribution from other Bragg spots, nondipole transitions or channeling effects[21]. Therefore, for each practical case, one need to find the exact collection semi-angle for the magic-angle condition in each specific STEM-EELS system. Consequently, for each convergence semi-angle (α) at the

objective aperture, there is a collection angle β, for which the core-edge shape becomes orientation independent. In our JEOL JEM-ARM200F setup (200 kV), the available and closest parameter setting to this ideal measurement condition is 7.3 mrad for β, under which a small variation of around 5% in the intensity of EELS C-*K* edge was achieved.

Supplementary References

1. Chen, X. & Kato, T. Growth mechanism and composition of ultrasmooth a-C:H:Si films grown from energetic ions for superlubricity. *J. Appl. Phys.* **115,** 044908 (2014).
2. Chen, X., Kato, T. & Nosaka, M. Origin of superlubricity in a-C:H:Si films: A relation to film bonding structure and environmental molecular characteristic. *ACS Appl. Mater. Interfaces* **6,** 13389-13405 (2014).
3. Bowden, F. P. & Tabor, D. *The Friction and Lubrication of Solids* (Clarendon Press, 1986).
4. Casiraghi, C., Ferrari, A. C. & Robertson, J. Raman spectroscopy of hydrogenated amorphous carbons. *Phys. Rev. B* **72**, 085401 (2005).
5. Ferrari, A. C. & Robertson, J. Interpretation of Raman spectra of disordered and amorphous carbon. *Phys. Rev. B* **61**, 14095-14107 (2000).
6. Egerton, R. F., Li, P. & Malac M. Radiation damage in the TEM and SEM. *Micro* **35**, 399-409 (2004).
7. Mølhave, K., Gudnason, S. B., Pedersen, A. T., Clausen, C. H., Horsewell, A. & Bøggild, P. Electron irradiation-induced destruction of carbon nanotubes in electron microscopes. *Ultramicroscopy* **279**, 34-42 (1996).
8. Wan, L. & Egerton, R. F. Preparation and characterization of carbon nitride thin films. *Thin Solid Films* **18**, 1987-1992 (2000).
9. Papworth, A. J., Kiely, C. J., Burden, A. P., Silva, S. R. P. & Amaratunga, G. A. J. Electron-energy-loss spectroscopy characterization of the sp^2 bonding fraction within carbon thin films. *Phys. Rev. B* **62**, 12628-12631 (2000).
10. Bernier, N., Bocquet, F., Allouche, A., Saikaly, W., Brosset, C., Thibault, J. & Charaï, A. A methodology to optimize the quantification of sp^2 carbon fraction from K edge EELS spectra. *J. Electron Spectrosc.* **164**, 34-43 (2008).
11. Berger, S. D., McKenzie, D. R. & Martin, P. J. EELS analysis of vacuum arc-deposited diamond-like films. *Philos. Mag. Lett.* **57**, 285-290 (1988).
12. Ferrari, A. C. *et al*. Density, sp^3 fraction, and cross-sectional structure of amorphous carbon films determined by x-ray reflectivity and electron energy-loss spectroscopy. *Phys. Rev. B* **62**, 11089-11103 (2000).
13. Zhang, X., Schneider, R., Müller, E. & Gerthsen, D. Practical aspects of the quantification of sp^2-hybridized carbon atoms in diamond-like carbon by electron energy loss spectroscopy. *Carbon* **102,** 198-207 (2016).
14. Ponsonnet, L. *et al*. EELS analysis of hydrogenated diamond-like carbon films. *Thin Solid Films* **319**, 97-100 (1998).

15. Galvan, D., Pei, Y. T., De Hosson, J. Th. M., & Cavaleiro, A. Determination of the sp^3 C content of a-C films through EELS analysis in the TEM. *Surf. Coat. Technol.* **200**, 739-743 (2005).
16. Hébert, C. Practical aspects of running the WIEN2k code for electron spectroscopy. *Micron* **38**, 12-28 (2007).
17. Nelhiebel, M., Louf, P.-H., Schattschneider, P., Blaha, P., Schwarz, K. & Jouffrey, B. Theory of orientation-sensitive near-edge fine-structure core-level. *Phys. Rev. B* **59**, 12807-12814 (1999).
18. Jouffrey, B., Schattschneider, P. & Hébert, C. The magic angle: a solved mystery. *Ultramicroscopy* **102**, 61-66 (2004).
19. Daniels, H., Brown, A., Scott, A., Nichells, T., Rand, B. & Brydson, R. Experimental and theoretical evidence for the magic angle in transmission electron energy loss spectroscopy. *Ultramicroscopy* **96**, 523-534 (2003).
20. Schattschneider, P., Hébert, C., Franco, H. & Jouffrey, B. Anisotropic relativistic cross sections for inelastic electron scattering, and the magic angle. *Phys. Rev. B* **72**, 045142 (2005).
21. Hébert, C., Schattschneider, P., Franco, H. & Jouffrey, B. ELNES at magic angle conditions. *Ultramicroscopy* **106**, 1139-1143 (2006).
